# Two spatiotemporally distinct value systems shape reward-based learning in the human brain

**DOI:** 10.1038/ncomms9107

**Published:** 2015-09-08

**Authors:** Elsa Fouragnan, Chris Retzler, Karen Mullinger, Marios G. Philiastides

**Affiliations:** 1 Institute of Neuroscience & Psychology, University of Glasgow, 58 Hillhead Street, Glasgow G12 8QB, UK; 2 Department of Behavioural & Social Sciences, University of Huddersfield, Huddersfield HD1 3DH, UK; 3 Sir Peter Mansfield Magnetic Resonance Center, School of Physics and Astronomy, University of Nottingham, Nottingham NG7 2RD, UK; 4 Birmingham University Imaging Centre, School of Psychology, University of Birmingham, Birmingham B15 2TT, UK

## Abstract

Avoiding repeated mistakes and learning to reinforce rewarding decisions is critical for human survival and adaptive actions. Yet, the neural underpinnings of the value systems that encode different decision-outcomes remain elusive. Here coupling single-trial electroencephalography with simultaneously acquired functional magnetic resonance imaging, we uncover the spatiotemporal dynamics of two separate but interacting value systems encoding decision-outcomes. Consistent with a role in regulating alertness and switching behaviours, an early system is activated only by negative outcomes and engages arousal-related and motor-preparatory brain structures. Consistent with a role in reward-based learning, a later system differentially suppresses or activates regions of the human reward network in response to negative and positive outcomes, respectively. Following negative outcomes, the early system interacts and downregulates the late system, through a thalamic interaction with the ventral striatum. Critically, the strength of this coupling predicts participants' switching behaviour and avoidance learning, directly implicating the thalamostriatal pathway in reward-based learning.

## Abstract (web summary)

Learning to reinforce rewarding decisions and avoiding repeated mistakes is critical, yet the neural systems mediating feedback processing in value-guided choices remain elusive. Here the authors uncover the spatiotemporal dynamics of two separate but interacting value systems during learning.

## Graphical abstract

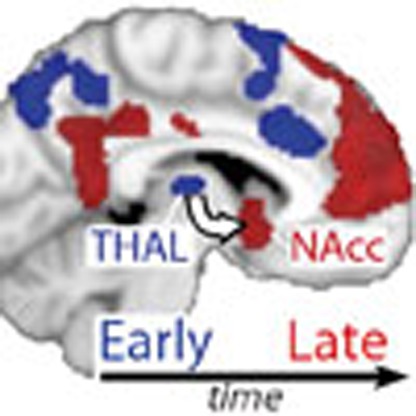

Imagine picking wild berries in a forest when suddenly a swarm of bees flies out from behind a bush. In a split second, your motor system has already reacted to flee the swarm. This automatic response constitutes a powerful survival mechanism that allows efficient behaviour switching to escape from a potential hazard in the environment. In turn, a separate and more deliberate process of learning to avoid similar situations will also occur, rendering future berry picking attempts less appealing.

The reinforcement sensitivity theory (RST) introduced by Jeffrey Gray^1^ in the 1970's was the first to describe two distinct decision-outcome value systems that trigger avoidance behaviour and orchestrate learning. According to RST, the first value system quickly assesses whether an outcome is positive or negative to alert an organism to take immediate action if required, while the second estimates all relevant information necessary to adjust future actions. In its initial form, the theory also postulated an interaction between the two systems such that the quick evaluation of outcome valence by the first system would modulate the second system to update future value expectations^1^.

To date, and despite RST's intuitive appeal, the biological validity and neural underpinnings of the two value systems (including their potential interactions) remain unclear. In line with RST, recent human electroencephalography (EEG) data revealed two temporally distinct processing stages of outcome value; an early valence-sensitive process thought to be driven by an automatic alertness response to negative outcomes^2^,^3^,^4^ and a later, more deliberate, assessment of the value information required for learning and updating reward expectations^3^. The poor spatial resolution of the EEG, however, precludes a thorough characterization of the spatial generators associated with each stage.

Conversely, functional magnetic resonance imaging (fMRI) studies investigating a pure categorical response to positive versus negative outcomes offer evidence of a distributed network of activations in response to outcome valence^5^,^6^,^7^,^8^,^9^,^10^. The low temporal resolution of the blood-oxygen-level-dependent (BOLD) signal, however, precludes a rigorous assessment of the relative timing and potential interactions between these activations. Here we combine single-trial EEG with simultaneously acquired fMRI to assign temporal order to these activations by mapping them onto the two valuation systems identified earlier using stand-alone EEG^3^. Our hypothesis is that endogenous trial-to-trial variability in the two temporally distinct EEG components can be used to form separate BOLD predictors (rather than using a categorical predictor representing outcome valence) to tease apart the cortical and subcortical networks associated with each system.

Separating these networks in time will also enable the investigation of potential interactions between the two value systems. We hypothesize that an early alertness system would likely engage autonomic arousal and motor-preparatory structures, whereas the late system would encompass regions more directly involved in reward processing. Relatedly, recent animal studies using optogenetics and electrophysiology have started to examine the functional role of the thalamostriatal pathway in mediating the interaction between these structures to exert control over learning-related plasticity^11^,^12^. To date, most animal and human neuroimaging studies have largely overlooked this pathway and focused instead on the connections between the dopaminergic system in the midbrain and its direct projection sites in the striatum (STR) and prefrontal cortex^13^,^14^.

Here, in line with our hypotheses, we uncover two spatiotemporally distinct but interacting outcome value systems associated with learning in the human brain. We show that an early system initiates a fast alertness response in the presence of negative outcomes, while a later system controls reward learning and value updating. Moreover, we show that the early system downregulates the late system to promote avoidance learning via a thalamostriatal interaction, imposing new constraints on theories of reward processing and outcome evaluation.

## Results

**Figure 1:**
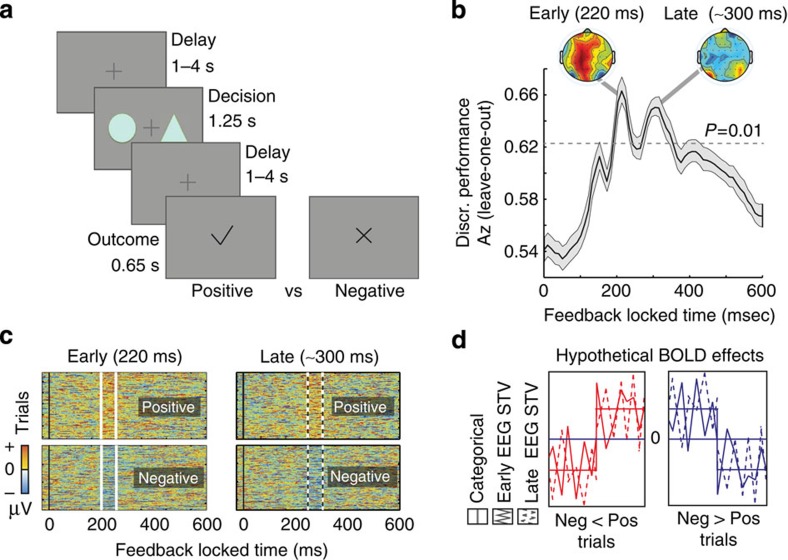
Experimental design and temporal characterization of separate outcome value systems. (**a**) Schematic representation of the experimental paradigm. On each trial, two abstract symbols (selected from a larger set of three symbols) were presented for a maximum of 1.25 s. During this time, subjects had to select, by pressing one of two buttons, the symbol that was most likely to lead to a reward. Once a decision was made, a random delay was presented before the outcome was revealed. A tick and a cross were used to inform the participants of a positive (constant reward) and a negative (non-rewarding) outcome, respectively. Participants (*n*=20) performed 2 blocks of 170 trials each. (**b**) Multivariate discriminator performance (*A*_*z*_) during positive versus negative outcome discrimination of outcome-locked EEG responses, averaged across subjects. Shaded error bars represent s.e. across subjects. The dotted line represents the average *A*_*z*_ value leading to a significance level of *P*=0.01, estimated using a bootstrap test. Two outcome value components (Early and Late) were revealed, with spatially distinct scalp topographies as estimated at time of maximum discrimination. (**c**) Single-trial discriminant component maps, for a representative subject. The four panels represent the discriminator amplitudes for the Early and Late components for positive and negative outcome trials using the training windows shown by the vertical white bars (solid: Early, dashed: Late). (**d**) Hypothetical value-related BOLD effects showing either greater overall BOLD signal for positive than negative outcomes and *vice versa* (red and blue curves, respectively). Three different BOLD predictors were used to model these effects: a conventional categorical regressor for positive versus negative outcomes and two parametric regressors modulated by the single-trial variability (STV) in the discriminator amplitudes of positive and negative outcomes in each of the Early and Late EEG components (extracted from subject-specific windows corresponding to the two components—solid and dashed windows, as seen in **c**).

We collected simultaneous EEG–fMRI data from 20 participants while they performed a probabilistic reversal-learning task^3^,^8^. On each trial, subjects saw a pair of abstract symbols and through feedback learned to select the symbol with the highest reward probability. On reaching a predefined performance criterion, the high reward probability was re-assigned to a different symbol in the set and subjects had to enter a new learning phase (that is, a ‘reversal' in reward contingencies was introduced; Fig. 1a). Overall subjects achieved multiple reversals (20.4±2.1, see Supplementary Note 1) during the course of the experiment suggesting a high degree of engagement with the task. Overall, participants' responses were probabilistic based on expected values assigned to each symbol on individual trials, in line with the principles of a simple reinforcement-learning mechanism (see Supplementary Methods).

### Two temporally specific components of outcome value

To identify temporally distinct neuronal components associated with outcome value, we used single-trial multivariate discriminant analysis on EEG signals locked to the delivery of the decision-outcome^15^. Specifically, for each participant, we estimated linear weighting of the EEG electrode signals (that is, spatial filters) that maximally discriminated between positive versus negative outcomes over several temporally distinct training windows (equation (1)). Applying the estimated spatial filters to single-trial data produced a measurement of the resultant discriminating component amplitudes, which we later used to parametrically modulate the amplitude of fMRI regressors (Supplementary Fig. 1). These values represent the distance of individual trials from the discriminating hyperplane and can be thought of as a surrogate for the neuronal response variability following positive and negative outcomes, with activity common to both conditions removed^15^,^16^,^17^,^18^. Our discriminator was trained to map positive component amplitudes to positive outcomes and negative component amplitudes to negative outcomes.

To quantify the discriminator's performance over time we used the area under a receiver operating characteristic curve (that is, *A*_*z*_ value) with a leave-one-out trial cross validation approach. Using this method, we identified two temporally distinct EEG components discriminating between positive and negative outcomes: an Early component peaked, on average, 219 ms following the outcome whereas a Late component peaked, on average, at 308 ms (Fig. 1b). Importantly, both components were present in each individual participant (Supplementary Fig. 2a and Supplementary Note 2), confirming our EEG data was of sufficiently high quality after removal of MR-related artifacts (see Methods). Control analyses revealed that neither of these components arose due to outcome salience^19^ (that is, the deviation from expectations estimated with a classical reinforcement-learning model, Supplementary equations (1–3), Supplementary Fig. 2b and Supplementary Note 3), the contextual sequence of outcomes^20^ (that is, the ratio of positive versus negative outcomes; Supplementary Fig. 2c and Supplementary Note 4), or differences in the visual properties of the outcome stimuli (Supplementary Fig. 2d and Supplementary Note 5).

Moreover, scalp topographies (Fig. 1b, equation (2)) revealed broad and largely distinct spatial profiles for the two components, providing initial support for the presence of separate generators associated with each component. Furthermore, trial-by-trial amplitude variations in the two discriminating components were largely uncorrelated (*r*=0.09*, P*=0.35 and *r*=0.17*, P*=0.24 for positive and negative outcomes, respectively). Taking advantage of the latter, we used the endogenous single-trial variability (STV) in the component amplitudes (as highlighted in Fig. 1c for one participant) to build two parametric EEG-informed fMRI regressors to identify the brain networks correlating with each of the Early and Late outcome value components.

Specifically, we built a general linear model (GLM) designed to investigate the extent to which the BOLD signal across the whole brain correlated with the EEG STV associated with each component either positively or negatively (that is, revealing regions activated more for positive compared with negative outcomes and *vice versa*; Fig. 1d). Note that while deeper/subcortical structures contribute less to the EEG signal, our method can still expose these regions through correlations with the cortical sources of the EEG STV. For comparison, we also used a separate GLM in which we introduced a single categorical BOLD predictor for outcome value (Fig. 1d), as was previously done in stand-alone fMRI studies^5^. In both models, we included a separate parametric regressor to absorb any unaccounted variance in the degree of outcome salience (Supplementary Fig. 3 and Supplementary Note 6; see Methods for full design details).

### Conventional fMRI of outcome value

**Figure 2:**
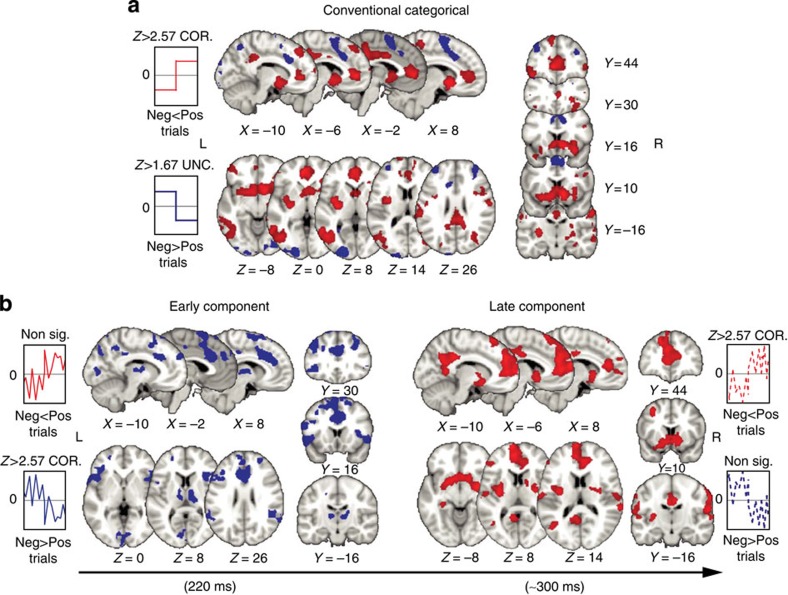
Spatiotemporal characterization of the Early and Late value systems. (**a**) A distributed network of activations including areas showing greater BOLD response for positive than negative outcomes (red clusters, mixed-effects (*n*=20), *Z*>2.57, corrected) and areas showing greater BOLD response for negative than positive outcomes, *albeit* at a more lenient threshold (blue clusters, mixed-effects (*n*=20), *Z*>1.67 uncorrected) using a conventional categorical outcome regressor (Supplementary Table 1). (**b**) A parametric regressor, based on the EEG STV in the Early value component, absorbed all activations that appeared in the conventional analysis in (**a**) exhibiting greater response for negative compared with positive outcomes (blue clusters) and additional unique activation clusters showing the same overall response profile (Neg>Pos; Supplementary Table 2). A parametric regressor based on the EEG STV in the Late value component, absorbed exclusively the activations that exhibited greater response for positive compared with negative outcomes in the conventional analysis in (**a**) (red clusters), including additional unique clusters showing the same overall response profile (Pos>Neg; Supplementary Table 2). All activations represent mixed-effects (*n*=20) and are rendered on the standard MNI brain at *Z*>2.57, corrected using a resampling procedure (minimum cluster size=76 voxels; see Methods).

Our conventional fMRI analysis using a single categorical outcome regressor (GLM 1; Methods) revealed a distributed network of activations including areas showing greater BOLD response for positive than negative outcomes (Pos>Neg; Fig. 2a, red clusters) and areas showing the opposite effect (Neg>Pos; Fig. 2a, blue clusters). Regions in which the BOLD signal was greater for positive than negative outcomes included areas of the human reward network^5^,^6^,^8^,^9^,^21^, such as the ventromedial prefrontal cortex (vmPFC), the STR, the amygdala and the dorsal posterior cingulate cortex (dPCC). Regions in which the BOLD signal was greater for negative than positive outcomes were overall less statistically reliable (surviving only an uncorrected threshold) and included clusters in the anterior mid-cingulate cortex (aMCC; often also labelled as dorsal anterior CC), the supplementary motor area and dorsolateral prefrontal cortex bilaterally. Overall these results agree with the large body of literature reporting activations relating to the contrast between positive versus negative outcomes^5^,^6^,^10^ (see Supplementary Table 1 for whole-brain results).

### EEG–fMRI reveals early and late outcome value systems

Even though the conventional analysis revealed a distributed set of activations for both the positive versus negative contrast and *vice versa*, their relative timing and potential interactions remain unclear. The main goal of our EEG-informed fMRI analysis (GLM 2; Methods) was the assignment of temporal order to the fMRI activations identified above by characterizing the extent to which these could be explained by the Early and Late outcome value EEG components. In this analysis, we capitalized on the additional explanatory power afforded to us by the EEG STV (that is, endogenous variability) in each component, which ought to carry more information about the internal processing of decision-outcomes than the stable (categorical) representation of the external stimulus valence. Thus this approach could provide a full spatiotemporal characterization of the networks associated with outcome value, enable identification of latent brain states (unobservable in the conventional analysis) and offer mechanistic insights regarding the functional role of the relevant networks.

Critically, we only found negative correlations with the EEG STV in our Early value component, which absorbed virtually all activations that appeared in the conventional analysis, exhibiting greater response for negative compared with positive outcomes (Fig. 2b, left panel; Supplementary Table 2). In addition, we observed unique activation clusters (compared with the conventional analysis) in the centromedial thalamus (CM-THAL) bilaterally, the anterior insula (aINS), as well as along the posterior MCC, extending to the dPCC. These areas were significantly more activated compared with the conventional analysis (paired *t*-tests, all *P*<0.05). This further confirms that the endogenous variability in our electrophysiologically derived measure of outcome value carries additional explanatory power over and above its externally (stimulus) defined counterpart. Conversely, we only found positive correlations with the EEG STV in our Late value component, which absorbed exclusively the activations that exhibited greater response for positive compared with negative outcomes in the conventional analysis (Fig. 2b, right panel; Supplementary Table 2). We also found activations in the anteromedial and superior medial prefrontal cortices, as well as the ventral PCC that were absent from the conventional analysis. Direct comparisons between the EEG-informed and conventional analysis in these regions revealed significant differences (paired *t*-tests, all *P*<0.05), highlighting the importance of exploiting the EEG STV to reveal latent brain states.

Taken together, our results paint a striking spatiotemporal picture of the underlying network. Specifically, our Early value component arises from a network of regions implicated in generating states of autonomic arousal that control immediate behavioural responses, as well as adjustment and negative outcome processing^22^,^23^,^24^. In contrast, our Late value component is linked to brain regions that play a crucial role in reward processing and value-guided learning^9^,^21^,^25^. Accordingly, our findings appear consistent with our original two separate value systems hypothesis, whereby an early automatic alertness response to outcome valence is followed by a later process involved in updating value information and guiding future behaviours. This interpretation is supported further by evidence that the Early system predicts response caution following negative outcomes, while the Late system predicts value updating after each outcome (see Supplementary Note 7).

### Early and late responses to positive and negative outcomes

Thus far, we demonstrated how the two value systems respond differentially across positive and negative outcomes (that is, overall Neg>Pos for the Early system and Pos>Neg for the Late one). However, the extent to which positive and negative outcomes could separately explain the BOLD responses associated with each of the two systems remains unclear as conventional fMRI studies using a categorical predictor of outcome valence can only capture relative changes across conditions. Here we capitalized instead on the endogenous trial-by-trial variability in response to identical outcomes (that is, either rewarded or non-rewarded) to understand how the Early and Late systems respond separately to positive and negative outcomes.

**Figure 3:**
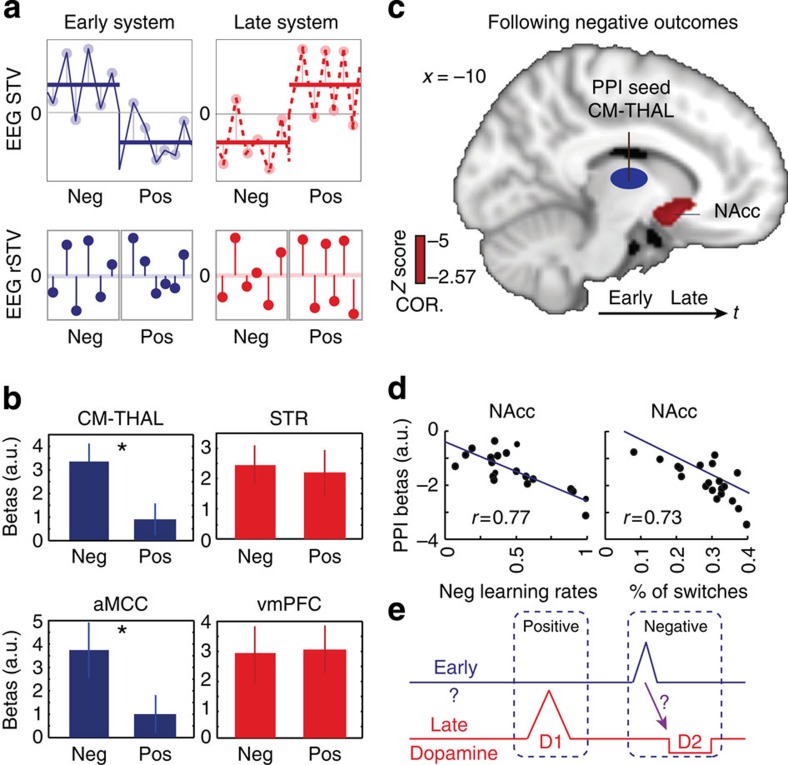
Separate responses of the Early and Late systems to negative and positive outcomes and their interaction. (**a**) Demeaning the EEG STV for each value system and outcome type separately (top panels) produced trial-to-trial residual fluctuations (EEG rSTV) in which the overall contribution of the categorical value contrast was removed (bottom panels). This manipulation introduced four new fMRI regressors to examine the extent to which negative and positive outcomes could explain the BOLD responses associated with each of the Early and Late systems separately. (**b**) The regions of the Early system correlated with the residual fluctuations related to negative outcomes only (that is, higher EEG rSTV leading to higher BOLD). Group regression coefficients from the CM-THAL and aMCC are shown for illustration (Supplementary Tables 3 and 4). Direct comparisons revealed significant differences in the response profile between positive and negative outcomes. In contrast, regions of the Late system correlated with the residual fluctuations in both negative and positive outcomes. Group regression coefficients from the STR and vmPFC are shown for illustration (Supplementary Tables 3 and 4). Error bars represent s.e. across subjects. (**c**) The CM-THAL of the Early system exhibited a strong inverse coupling with a striatal cluster in the NAcc belonging to the Late system, following negative outcomes (*n*=20). The NAcc activation is shown at *Z*>2.57, *P*<0.05 corrected, on the standard MNI template. (**d**) Participants that exhibited stronger (more negative) thalamostriatal coupling and hence stronger downregulation of the NAcc showed a higher rate of switching behaviour following negative outcomes (*r*=0.73*; P*<0.001) and higher negative learning rates (*r*=0.77*; P*<0.001), estimated using a classical reinforcement-learning model. (**e**) Graphical illustration of the two outcome value systems. Our data suggests that controlling reward learning might extend beyond the direct influence of the dopaminergic system, though future work would be required to elucidate the specific neuromodulatory pathways driving the two systems and their interactions.

Specifically, we demeaned the EEG STV for each system and outcome type separately to obtain trial-to-trial residual fluctuations (that is, EEG rSTV as illustrated in Supplementary Fig. 1c) in which the overall contribution of the categorical value contrast and any task-independent baseline effects were removed. We used these endogenous fluctuations to build four new parametric fMRI regressors in a new GLM analysis (Fig. 3a, EarlyNeg, EarlyPos, LateNeg and LatePos, GLM 3; Methods). We hypothesize that regions responding to each outcome type separately should continue to covary with the EEG rSTV in the relevant regressors above.

Interestingly, we found that regions of the Early system correlated with the endogenous variability related to negative outcomes only (that is, higher EEG rSTV leading to higher BOLD), including major clusters in the CM-THAL and aMCC reported earlier (Fig. 3b left, Supplementary Tables 3 and 4). This result suggests that the Early system is primarily activated by negative events. This is consistent with previous reports implicating the thalamo-cingulate pathway in avoidance control by alerting an organism of non-rewarding or undesirable outcomes and re-orienting behaviour towards alternative actions^23^,^26^,^27^. In contrast, regions associated with the Late system correlated significantly with the endogenous variability resulting from both negative and positive outcomes (that is, for negative outcomes: smaller EEG rSTV leading to lower BOLD; for positive outcomes: higher EEG rSTV leading to higher BOLD), including prominent activations in the STR and vmPFC (Fig. 3b right, Supplementary Table 3). This finding indicates that the Late system suppresses or activates these regions in response to negative and positive outcomes, respectively, an activity pattern consistent with the role of the dopaminergic system in motivating both avoidance and approach learning^9^,^14^.

### Early and late system interaction mediates learning

Having established the presence of two separate value systems, with distinct outcome-related response profiles, we turned to the question of whether the Early (alertness) system interacts with the Late (reward related) system to aid learning to avoid choices that previously led to negative outcomes, as proposed by the original RST^1^. To quantify potential interactions, we adopted a connectivity approach using a psychophysiological interaction analysis (PPI)^28^. As a seed region for the PPI analysis, we selected the CM-THAL for three main reasons: (1) the CM-THAL is one of the most prominent activations uniquely correlating with the EEG STV in our Early value component, (2) recent animal studies suggested that the CM-THAL exerts state-control over learning-related plasticity^11^,^12^ and (3) the CM-THAL is a major hub with strong connections to regions appearing in both the Early and Late systems^23^,^27^,^29^. We designed the PPI analysis to identify brain areas in the Late system that increase their connectivity with the thalamus following negative outcomes (see Methods).

This connectivity analysis revealed a significant inverse coupling between the thalamus and the ventral STR cluster we found in the Late system, which corresponds to the nucleus accumbens (NAcc), a known projection site of the dopaminergic system^13^ (Fig. 3c). Specifically, as the thalamic response in the Early system increased following negative outcomes, NAcc activity in the Late system decreased. The relative timing of these activations as captured by the EEG suggests that the interaction proceeds from the CM-THAL (Early) to the NAcc (Late). We further confirmed the directionality of this interaction using dynamic causal modelling analysis^30^,^31^ (Supplementary Fig. 4a and Supplementary Methods). Interestingly, this coupling was not evident in the EEG signal itself likely because these regions form only a small subset of the overall activations associated with each system, highlighting the complementary nature and the importance of integrating the two neuroimaging modalities.

The dynamics of this thalamostriatal inverse coupling is consistent with a mechanism of value updating, which in turn can alter future choice behaviour^27^,^32^,^33^,^34^,^35^. To test this interpretation and establish a direct link between the strength of this coupling and participants' behaviours, we performed an additional analysis. Specifically, we correlated the strength of the thalamostriatal coupling (regression coefficient from the PPI analysis) from each participant with individual switch patterns (fraction of switches following a negative outcome) and with learning rates associated with negative outcomes^33^ (as estimated with a classical reinforcement-learning model, Supplementary equations (1–3)). We hypothesized that those individuals exhibiting stronger (more negative) thalamostriatal coupling would be showing a higher rate of switching behaviour and, correspondingly, would be weighing recent negative outcomes more strongly (that is, show a higher learning rate in the model). Our findings confirmed this hypothesis (Fig. 3d), showing that the strength of the thalamostriatal coupling was a significant predictor of behavioural switches and learning rates (*P*<0.001 and *P*<0.001, respectively). The strength of this coupling remained a significant predictor of behaviour even after accounting for the individual activity of the CM-THAL and the NAcc (*P*=0.0045 and *P*=0.0019, for behavioural switches and learning rates, respectively). These findings offer the first instance in the human brain where the thalamostriatal pathway is directly linked to switching behaviour and updating value expectations in line with animal literature^12^,^36^.

Finally, we also looked at whether the CM-THAL covaried positively with other regions within the Early system itself and found that it was functionality connected to the aMCC and the aINS (Supplementary Fig. S3b), consistent with known connectivity patterns between these regions^37^,^38^. Repeating the PPI analysis with either the aMCC or aINS as seeds confirmed this connectivity profile within the Early system. Interestingly, however, only the CM-THAL showed a significant inverse coupling with the Late system as discussed above. These findings suggest that following negative outcomes, the CM-THAL interacts both with structures controlling early autonomic responses, as well as those activated later to update value information, acting as a major hub between the Early and Late systems^37^,^38^.

## Discussion

Here we integrated EEG and fMRI data by exploiting the trial-by-trial variability in the two neuroimaging modalities to provide a characterization of the global network dynamics associated with outcome value during reward-based learning in humans. Correlating electrophysiological and haemodynamic measures allowed ‘static' fMRI activations (resulting from temporal averaging and the slow dynamics of conventional fMRI) to be absorbed by temporally specific components of outcome value. This in turn offered temporal order to the underlying networks and enabled a rigorous characterization of relevant network interactions.

This approach led to the identification of two separate but interacting neural value systems associated with learning in the human brain. More specifically, our data suggests that a fast (Early) system processes mainly negative decision-outcomes and appears to serve a dual role. Specifically, it appears to initiate a fast alertness response in the presence of negative outcomes, while in parallel downregulates the response profile of a slower (Late) reward-related system to promote avoidance learning. Conversely, positive decision-outcomes primarily activate the brain network associated with the Late system, consistent with a role in approach learning and value updating, without a corresponding contribution from the Early system. The presence of these separate value systems suggests that different neurotransmitter pathways might modulate each system and facilitate their interaction (see illustration in Fig. 3e).

The brain regions associated with the Early system, such as the CM-THAL, the aMCC and neighbouring premotor regions, are known target sites of ascending noradrenergic and serotonergic projections, from the locus coeruleus and the raphe nucleus, respectively, that regulate alertness responses^39^. Although largely speculative, this observation indicates a possible role of these pathways in modulating the activity of the Early system, which appears to act as an ‘interrupt' signal of on-going activity in the Late system to first address an immediate challenge in the environment^35^. This idea is supported further by evidence showing that the onset time of the Late system (in the EEG) shifts later in time with the strength of the Early system (Supplementary Fig. 4b and Supplementary Note 8). Moreover, the profile of the Early EEG component is in line with the feedback-related negativity^40^,^41^, which was recently shown to respond to serotonergic rather than dopaminergic modulation^42^,^43^. Taken together, these findings suggest that the fast initial response of the Early system might not be facilitated by dopamine.

In contrast, the brain regions associated with the Late system (for example, vmPFC, STR, dPCC) have consistently been linked to the dopaminergic pathway^14^,^19^,^44^,^45^ and its role in learning. In particular, the incremental response profile we observed along the negative/positive outcome dimension (that is, decreases and increases in BOLD activity following negative and positive outcomes, respectively) is in line with the distinct roles of the D1 and D2 dopamine receptor subtypes, which have been shown to drive approach (D1 stimulation after a positive outcome) and avoidance learning (D2 suppression after a negative outcome), respectively^44^, in the appetitive domain. These findings also suggest that the Late EEG component, which is largely consistent with a P300-type evoked response referred to as feedback-related positivity^46^,^47^, could be under dopaminergic control, although this hypothesis remains to be tested.

Notably, recent evidence from animal electrophysiology suggests that the midbrain neurons mediating the avoidance learning highlighted above behave markedly different following negative outcomes depending on whether the outcome involved an omission of reward or a true loss/punishment^48^,^49^. Our work focuses on appetitive reinforcement and therefore positive and negative outcomes represent rewards and non-rewards, respectively. Whether or not our results extend to the aversive domain (for example, receiving punishments) remains unclear, though unpublished stand-alone EEG data from our lab using monetary gains and losses in an otherwise identical task yielded similar results (that is, an early and a late outcome value components).

Importantly, we also showed that the observed decrease of striatal activity in the Late system (NAcc), following negative outcomes is regulated by an increase in thalamic activity in the Early system (CM-THAL). Correspondingly, recent animal studies have suggested that a direct CM-THAL/NAcc interaction might play a major role in inhibiting the activity of the network involved in motivational learning^11^,^12^,^27^,^36^. In line with these animal studies, our work suggests that the Early value system exerts state-control over the Late system to promote switching behaviours and avoidance learning via a similar thalamostriatal pathway.

It has long been known that the STR, in particular the NAcc, receives glutamatergic inputs from the CM-THAL^50^,^51^, however, the functional role of this interplay in reward learning has long been neglected^13^,^14^. Importantly, the glutamatergic inputs in the NAcc have a reliable inhibitory effect on striatal cholinergic interneurons^51^ that in turn suppress D2 receptors in the STR^52^,^53^. One hypothesis could be that this thalamostriatal interplay is part of an extended circuitry including regions of the brainstem, such as the VTA, the primary source of dopamine-releasing neurons^13^, and medial prefrontal cortex that regulate negative reinforcement learning. Though this interpretation is still putative, we hope that future studies, including high-resolution fMRI of the brainstem^54^ and more invasive electrophysiological experiments, will elucidate the precise role of this neuromodulatory pathway and the interactions of the two value systems.

In conclusion, we demonstrated that capitalizing on the endogenous variability in electrophysiologically derived measures of outcome value, recorded simultaneously with fMRI, offered critical new insights, otherwise unobservable with each modality alone. As such our general research approach opens up new avenues for the investigation of the neural systems underlying reward-based decision making in humans. Crucially, our findings also have the potential to further improve our understanding of how everyday responses to rewarding or stressful events can affect our capacity to make optimal decisions, as well as facilitate the study of how mental disorders—such as chronic stress, obsessive-compulsive-disorder, post-traumatic disorder and depression—affect learning and strategic planning.

## Methods

### Participants

Twenty-four subjects participated in the experiment. Four were removed from the analysis for excessive head movements inside the scanner. The remaining 20 subjects (8 males), aged between 18–31 years (mean=21 years, s.d.±2.6), were included in all subsequent analyses. All were right handed, had normal or corrected-to-normal vision and reported no history of psychiatric, neurological or major medical problems, and were free of psychoactive medications at the time of the study. Written informed consent was obtained in accordance with the School of Psychology Ethics Committee at the University of Nottingham.

### Stimuli display

We used a set of 12 abstract symbols that were adapted from our previous experiment^3^. In addition to these symbols, we used a tick and a cross to provide positive and negative feedback, respectively. The stimuli (180 × 180 pixels), feedback symbols (125 × 125 pixels) and fixation cross (30 × 30 pixels) were equated for luminance and contrast. A Windows Professional 7, 64 bit-based machine (3 gb RAM) with an nVidia (Santa Clara, CA) graphics card and Presentation software (Neurobehavioral Systems Inc., Albany, CA) controlled the stimulus display. Images were projected with an EPSON EMP-821 projector (refresh rate: 60 Hz, resolution: 1280 × 1024 pixels) onto a screen which was 2.3 m from the subject (projection screen size: 120 × 90 cm). Stimuli and feedback symbols were subtended 4° × 4° and 3° × 3° of visual angle, respectively.

### Reversal-learning task

The experiment consisted of 2 blocks of 170 trials each (340 trials in total). The two blocks were separated by a break. At the beginning of each block, subjects were shown a screen with three symbols. For each block, a different triplet was chosen randomly from the larger set of 12 symbols. Subjects were told that their goal was to identify the symbol with the highest reward probability. They were also informed that in the course of each block, the highest reward probability might shift to one of the other two symbols and that they would have to adjust their choices accordingly. Each rewarded trial earned them 1 point, while unrewarded trials earned them 0 points. Subjects were also told that they would receive a fixed payment for participation (£15 per hour) and an additional amount (up to a maximum of £45) based on the outcome of a random subset of trials selected at the end of the experiment (excluding ‘lost' trials—see below). No further details regarding the mapping between earned points and the final payoff were given to the subjects.

Each trial began with the presentation of a central fixation cross for a random delay in the range 1–4 s (mean delay 2.5 s). To ensure alertness during the experiment and minimize saccades, subjects were instructed to focus on the central fixation. Two of the three symbols were then placed to the left and to the right of the fixation cross for 1.25 s. During this time, subjects had to choose one of the symbols by pressing the left or right button on a fORP MRI compatible response box (Current Design Inc., Philadelphia, PA, USA) using their right index or middle finger, respectively. When subjects indicated their choice, the fixation cross flickered for 100 ms to signal that the response was registered successfully. Next, the decision outcome was presented after a second random delay in the range 1–4 s (mean delay 2.5 s). Positive and negative outcomes were provided by placing a tick or a cross, respectively, in the centre of the screen for 650 ms. Trials, in which subjects failed to respond within the 1.25 s of the stimulus presentation, were followed by a ‘lost trial' message and were excluded from further analysis. Figure 1a summarizes the sequence of these events. To increase detection power and estimation efficiency in the fMRI analysis, the sequence of these events and the timing of the two delay periods were optimized using a genetic algorithm^55^,^56^.

At any one point in the course of the experiment, one of the three symbols was associated with a ‘high' reward probability of 0.7 (that is, good symbol) compared with the remaining two symbols (that is, bad symbols), each of which had a reward probability of 0.3. Participants were naive about the exact reward probabilities assigned to the symbols and they were told to learn to choose the good symbol through trial and error and by taking into account the decision-outcome on each trial. To detect when subjects learned to choose the symbols with the higher reward probability, we defined a learning criterion. Specifically, subjects were thought to have learned the good symbol when they chose it in five out of the last six trials. Every time the learning criterion was reached, a reversal was introduced by randomly changing the reward contingencies across the three symbols (that is, the ‘high' reward probability was re-assigned to a different symbol). To make reversals less predictable, we included additional trials (that is, buffer trials) after the learning criterion was reached that followed a Poisson process, such that there was a probability of 0.3 that a reversal took place on any given post-learning criterion trial (with a minimum of 1 and a maximum of 8 trials) and before participants entered a new learning phase.

To prevent subjects from searching for non-existent patterns and to reduce cognitive load, we presented the three possible pair combinations of the three symbols in a fixed order (that is, *AB*, *BC*, *CA*)—though the presentation side of the symbols on the screen (left or right) of the fixation cross was randomized. Subjects were explicitly informed about this manipulation. Another key component of this paradigm was that we presented stimulus pairs chosen from a pool of three symbols. This manipulation served two important purposes. First, it encouraged subjects to engage in an exploration phase to identify the most rewarding symbol after reversals occurred. Second, it forced the subjects to choose between the two least rewarding symbols (in every third trial, when the two were presented together) even when they had learned the task. Overall, when deciding between the two bad symbols subjects chose the one that carried the highest expected value as estimated based on past reward history (Supplementary Fig. 2b). This manipulation ensured a more balanced number of positive and negative outcomes.

### Training

Two weeks prior to the main experiment, participants were invited to complete a full set of trials on the main task. This training session was designed to familiarize participants with the task and identify those individuals that understood the probabilistic nature of the task, whom we invited back for the main experiments. The day of the simultaneous EEG-fMRI scanning session, prior to the main experiment, all subjects completed an additional 100 trials to remind them of the main task.

### Electrophysiological data acquisition

EEG was collected simultaneously with the fMRI data using an MR-compatible EEG amplifier system (BrainAmps MR-Plus, Brain Products, Germany) and recorded using Brain Vision Recorder (BVR; Version 1.10, Brain Products, Germany) with a 5-kHz sampling rate. Data underwent online (hardware) filtering with a band-pass filter of 0.016–250 Hz. The EEG cap consisted of a 64 Ag/AgCl scalp electrodes positioned according to the international 10–20 system of electrode positioning. Reference and ground electrodes were embedded in the EEG cap and placed along the midline (reference electrode: between electrode Fpz and Fz, ground electrode: between electrode Pz and Oz). Each electrode had in-line 10 kΩ surface-mount resistors to ensure subject safety. All leads were twisted for their entire length and bundled together to minimize inductive pick-up. All input impedances were kept below 20 kΩ (including the 10 kΩ surface-mount resistors on each electrode). Acquisition of the EEG data was synchronized with the MR data acquisition (Syncbox, Brain Products, Germany) and MR-scanner triggers were collected separately to enable offline removal of MR gradient artifacts. Scanner trigger pulses were lengthened to 50 μs using an in-house pulse stretcher to facilitate accurate capture by the BVR. Experimental event codes were also synchronized with the EEG data and collected using the BVR software.

To minimize the MR gradient artifacts, we ensured that electrodes Fp1 and Fp2 were at the isocentre of the MR scanner in the *z*-direction^57^ when placing participant's in the scanner. We achieved this, by aligning these two electrodes with the laser beam used to position the participants inside the bore. A 32-channel SENSE head coil incorporated an access port, which allowed the cables from the EEG cap to run along a straight path out of the scanner and helped to ensure there were no wire loops, minimizing the risk of RF heating of the EEG cap and associated cables and induce EEG artifacts. In addition, the cabling was isolated from scanner vibrations as much as possible to minimize induced artifacts, through the use of a cantilevered beam^58^.

### EEG pre-processing

We performed EEG pre-processing offline using Matlab (Mathworks, Natick, MA). EEG signals recorded inside an MR scanner are contaminated with gradient artifacts and ballistocardiogram (BCG) artifacts due to magnetic induction on the EEG leads. We first removed the gradient artifacts. Specifically, from each functional volume acquisition we subtracted the average artifact template constructed using the 80 volumes centred on the volume-of-interest using in-house Matlab software. We repeated this process for as many times as there were functional volumes in our data sets. We subsequently applied a 10-ms median filter to remove any residual spike artifacts. Next, we removed standard EEG artifacts by applying a 0.5-Hz high-pass filter to remove DC drift, 50 and 100 Hz notch filters to remove electrical line noise, and 100 Hz low-pass filter to remove high frequency artifacts not associated with neurophysiological processes. These filters were applied together, non-causally to avoid distortions caused by phase delays.

BCG artifacts share frequency content with the EEG and as such are more challenging to remove. Here to avoid loss of signal power in the underlying EEG, we adopted a conservative approach based on our previous work^59^,^60^. Specifically, we only removed a small number of subject-specific BCG components using principal component analysis (see below) and relied instead on our single-trial classifiers (see single-trial EEG analysis section) to identify discriminating components that are likely to be orthogonal to the BCG. Note that this approach is robust to the presence of BCG artifact residuals due, specifically, to the multivariate nature of our classification techniques. BCG principal components were extracted from the data after the data were first low-pass filtered at 4 Hz to extract the signal within the frequency range where BCG artifacts are observed, and then subject-specific principal components (average number of components across subjects: 2.3) were determined. The sensor weightings corresponding to those components were projected onto the broadband data and subtracted out.

### Eye-movement artifact removal

Prior to the main experiment, we asked our participants (while in the scanner) to complete an eye-movement calibration task during which they were instructed to blink repeatedly on the appearance of a fixation cross in the centre of the screen and then to make several horizontal and vertical saccades according to the position of the fixation cross. The fixation cross subtended 0.6° × 0.6° of visual angle. Horizontal saccades subtended 30° and vertical saccades subtended 22°. This exercise enabled us to determine linear EEG sensor weightings corresponding to eye blinks and saccades (using principal component analysis) such that these components were projected onto the broadband data from the main task and subtracted out^61^.

### Single-trial EEG analysis

We applied a linear multivariate classifier to EEG data locked to the time of decision outcome, using the sliding window method in refs 15, 16, 17, 18. Specifically, we found a projection of the multidimensional EEG signal, ***x***_*i*_(*t*), where *i*={1…*T*} and *T* is the total number of trials, within a short time window that achieved maximal discrimination between positive and negative outcome trials. All time windows had a width of *N*=60 ms and the window centre *τ* was shifted from −100 to 600 ms relative to outcome onset, in 10-ms increments. We used a regularized Fisher discriminant analysis (see below for details)^62^ to learn the spatial weighting, ***w***(*τ*), that maximally discriminated between positive and negative outcomes, arriving at the one-dimensional projection *y*_*i*_(*τ*), for each trial *i* and a given window *τ*:


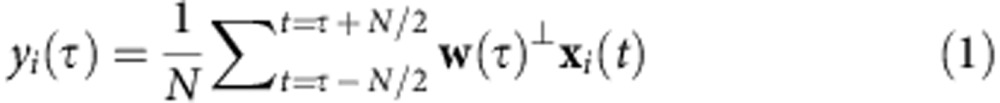


where *y*_*i*_(*τ*), is organized as a vector of single-trial discriminator amplitudes (1 × Trials), the spatial filter, ***w***(*τ*), is organized as a vector with as many weights as there are channels in the data (1 × 64) and data, ***x***_*i*_(*τ*), is organized as a matrix, with dimensions (64 × Trials/Samples). is used to indicate a transpose operator. We adopted this approach to identify all time windows *τ* yielding significant discrimination performance in the outcome period and used the resultant single-trial component amplitudes, *y*_*i*_(*τ*), to construct parametrically modulated BOLD predictors for our fMRI analysis as discussed below (see fMRI analysis section). Note that in separating the two groups of trials, the classifier was designed to map positive and negative discriminant component amplitudes to positive and negative outcomes, respectively. As such brain regions in the fMRI that correlated positively with the EEG STV showed an overall stronger response to positive rather than negative outcomes, whereas regions that correlated negatively showed the opposite effect (that is, stronger response to negative rather than positive outcomes).

The projection vectors ***w*** at each time window *τ* were estimated as: ***w***=***S***_**c**_(***m***_2_–***m***_1_) where ***m***_***i***_ is the estimated mean of condition *i* and ***S***_**c**_=1/2(***S***_**1**_+***S***_**2**_) is the estimated common covariance matrix (that is, the average of the condition-wise empirical covariance matrices, 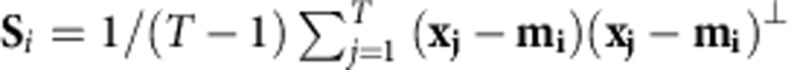, with *T*=number of trials). To treat potential estimation errors, we replaced the condition-wise covariance matrices with regularized versions of these matrices: 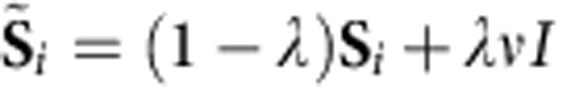, with *λ*∈[0, 1] being the regularization term and *ν* the average eigenvalue of the original ***S***_***i***_ (that is, trace(***S***_***i***_)/64). Note that *λ*=0 yields unregularized estimation and *λ*=1 assumes spherical covariance matrices. Here we optimized *λ* for each participant using a leave-one-out trial cross validation procedure (*λ*'s, mean±s.e.: 0.028 ±0.05) across the entire post-outcome period.

We quantified the performance of the discriminator for each time window using the area under a receiver operating characteristic curve, referred to as an *A_z_* value, using a leave-one-out trial procedure^63^. To assess the significance of the discriminator, we used a bootstrapping technique where we performed the leave-one-out test after randomizing the trial labels. We repeated this randomization procedure 1,000 times to produce a probability distribution for *A_z_*, and estimated the *A_z_* leading to a significance level of *P*<0.01. In addition, we implemented a separate temporal-clustering procedure using a similar randomization test. Specifically, we repeated the procedure above, each time identifying the maximum number of continuous time steps surviving the *A_z_* significance threshold found with the original bootstrapping technique described above. This in turn enabled us to produce a null distribution for the maximum number of continuous temporal windows and estimate a temporal cluster size leading to a significance level of *P*<0.05 (individually for each participant, average temporal cluster threshold: 4.7 time steps±2.1).

Given the linearity of our model, we also computed scalp topographies of the discriminating components resulting from equation (1) by estimating a forward model as:


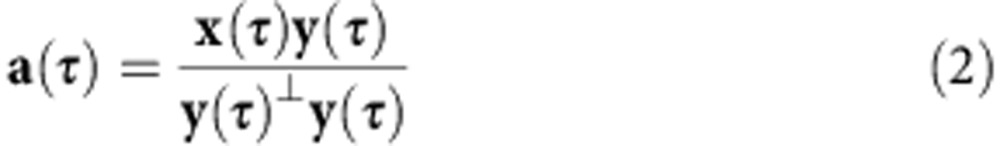


where *y*_*i*_(*τ*) is now shown as a vector ***y***(***τ***), where each row is from trial *i*, and ***x***_***i***_(*τ*) is organized as a matrix, ***x***(***τ***), where rows are channels and columns are trials, all for time window *τ*. These forward models can be viewed as scalp plots and interpreted as the coupling between the discriminating components and the observed EEG^15^,^17^,^59^. Code for the linear discriminant analysis described above is available at: http://liinc.bme.columbia.edu/downloads/lr1.2_plugin.tar.gz

To visualize the temporal profile of the resultant discriminating components across individual trials, we also constructed discriminant component maps (as seen in Fig. 1c). To do so, we applied the spatial weighting vectors, ***w***(*τ*) from a time window, *τ*, which led to significant discrimination performance between positive versus negative outcomes, to an extended time window (100 ms before until 600 ms after the outcome). Each row of one such discriminant component map represents a single trial across time (see, for example, Fig. 1c).

### MRI data acquisition

BOLD data sets were acquired on a 3 T Philips Achieva MRI scanner (Philips, Netherlands). Functional Echo-Planar-Imaging (EPI) data were acquired using an 32-channel SENSE head coil with SENSE factor 2.3 with an anterior–posterior fold over direction, 40 slices of 68 × 68 voxels with in-plane resolution of 3 × 3 mm and slice thickness of 3 mm and a flip angle of 80°. Repetition time (TR) was 2.5 s with an echo time (TE) of 40 ms. Slices were acquired in an interleaved order. In total, two separate runs of 468 volumes each were acquired corresponding to the two blocks of trials in the main experimental task. Anatomical images were acquired using a MPRAGE T1-weighted sequence that yielded images with a 1 × 1 × 1 mm resolution (160 slices of 256 × 256 voxels; TR: 8.2 ms, TE: 3.7 ms). A B0 map was acquired using a multi-shot gradient echo sequence with TE=2.3 ms and delta TE=5 ms with 3-mm isotropic resolution, 68 × 68 × 32 matrix, TR 383 ms, flip angle 90°, which was subsequently used to correct for distortion of the EPI data due to B0 inhomogeneities for each participant.

### fMRI pre-processing

The first five volumes from each fMRI run (pre-task period) were discarded to ensure a steady-state MR signal, and the remaining 463 volumes were used for the statistical analysis. Initial fMRI data pre-processing was performed using the FMRIB's Software Library (Functional MRI of the Brain, Oxford, UK) and included head motion correction, slice-timing correction, high-pass filtering (>100 s), and spatial smoothing (with a Gaussian kernel of 8 mm full-width at half maximum). Registration of EPI images to standard space (Montreal Neurological Institute, MNI) was performed using FMRIB's Non-linear Image Registration Tool with a 10-mm warp resolution^64^. The registration procedure involved transforming the EPI images into an individual's high-resolution space (with a linear six-parameter rigid body transformation) prior to transforming to standard space. Finally, we performed B0 unwarping to correct for signal loss and geometric distortion due to B0 field inhomogeneities in the EPI images^65^.

### fMRI analysis

Whole-brain statistical analyses of functional data were performed using a multilevel approach within the framework of a GLM, as implemented in FSL (using the FEAT module^66^):


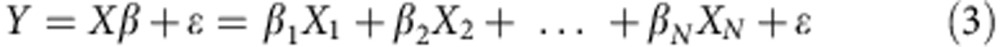


where *Y* is a *T × 1* (*T* time samples) column vector containing the times series data for a given voxel, and *X* is a *T* × *N* (*N* regressors) design matrix with columns representing each of the psychological regressors convolved with a canonical haemodynamic response function (double-γ function). *β* is a *N* × *1* column vector of regression coefficients (commonly referred to as betas or parameter estimates) and *ɛ* a *T* × *1* column vector of residual error terms.

A first-level analysis was performed to analyse each subject's individual runs, which were then combined using a second-level analysis (fixed effects). Finally, to combine data across subjects a third-level, mixed-effects model was used (FLAME 1), treating participants as a random effect. Time-series statistical analysis was carried out using FMRIB's improved linear model with local autocorrelation correction^67^. In total, we performed three different GLM analyses using this framework (see below).

### Conventional fMRI analysis of outcome value—GLM 1

We first ran a conventional fMRI analysis designed to identify the brain networks responding differentially to positive and negative outcomes using a simple categorical regressor for outcome valence. Specifically, locking at the time of outcome (that is, when the tick/cross appeared) we included four boxcar regressor with a duration of 100 ms for each regressor event: (1) an unmodulated regressor (all event amplitudes set to 1); (2) a simple categorical regressor for outcome valence (amplitudes set to +1 for positive and −1 for negative outcomes); (3) a fully parametric regressor whose event amplitudes were modulated by the unsigned prediction error (PE) estimates from a RL model (to control for salience effect) and (4) an unmodulated regressor for all lost trials. In addition we included an unmodulated regressor of no interest at the time of stimulus presentation (that is, decision phase) and six nuisance regressors, one for each of the motion parameters (three rotations and three translations).

### EEG-informed fMRI analysis of outcome value—GLM 2

In this analysis, we capitalized on the EEG STV in two highly discriminating components of outcome value (Fig. 1b; Early and Late). Specifically, we used the resulting trial-by-trial amplitude estimates of *y*_*i*_(*τ*) (equation (1)) for each component to build two separate BOLD predictors (Supplementary Fig. 1). Our hypothesis is that the endogenous trial-by-trial variability in these two components carries more information about the internal processing of decision-outcomes than the stable (categorical) representation of the external stimulus valence (in GLM 1). As such this approach could enable both separation of the relevant fMRI activations (as seen in the conventional analysis above), identification of latent brain states (activations unobserved in the conventional analysis) and assignment of temporal order to the underlying networks. We therefore replaced the categorical valence regressor in the conventional analysis above (GLM 1) with two fully parametric regressors modulated by the EEG STV in each of the Early and Late discriminating components of outcome value. We set the onset time of these regressors at the time of outcome. Shifting these to the actual times of the Early and Late components (as seen in the EEG) yielded identical results due to the sluggish nature of the haemodynamic response function. Dissociating the contribution of the two components was driven exclusively by amplitude modulation of our regressor events. The rest of the design was identical to GLM 1. To account for the shared variance between the two EEG-informed regressors, we also performed two supplementary analyses. Specifically, we repeated GLM 2 while orthogonalizing the regressor for the Early EEG component with respect to the one for the Late EEG component and *vice versa*. We found that in both designs, the activations correlating with the Early and Late components remained identical to those in the original model (See Supplementary Note 9).

### EEG-informed fMRI valence analysis—GLM 3

Demeaning the EEG STV for each value system and outcome type separately produced trial-to-trial residual fluctuations (EEG rSTV) in which the overall contribution of the categorical value contrast was removed (Supplementary Fig. 1c). This manipulation introduced four new fMRI regressors (Fig. 3a, EarlyNeg, EarlyPos, LateNeg and LatePos) to examine the extent to which negative and positive outcomes could explain the BOLD responses associated with each of the Early and Late systems (as identified in GLM 2) separately. The main motivation for this analysis rests with the idea that regions responding to each outcome type separately should continue to covary with the EEG rSTV (that is, electrophsyiologically derived endogenous variability) in the relevant regressors above. We therefore replaced the categorical valence regressor in the conventional analysis above (GLM 1) with four fully parametric regressors modulated by the EEG rSTV as described above. The rest of the design was identical to GLM 1/2.

The three GLM models highlighted above were selected to offer a hierarchically principled approach to illustrate what can be gained when the analysis proceeds from using a conventional (categorical) fMRI contrast (GLM 1), to using multiple single-trial EEG-informed predictors to absorb the activations appearing in the conventional analysis and offer temporal order to the relevant networks (GLM 2), to finally showing how the temporally specific activations identified in the previous step respond separately to positive and negative outcomes (GLM 3).

### Resampling procedure for fMRI thresholding

In order to properly correct the fMRI statistical maps for multiple comparisons, we used a resampling procedure that took into account the *a priori* statistics of the trial-to-trial variability in all of our fully parametric regressors (that is, EEG-derived regressors and model-based unsigned PE regressor) in a way that trades off cluster size and maximum voxel Z-score^68^. Specifically, we maintained the overall distributions of the EEG discriminating components (*y*_*i*_(*τ*) values for the Early and Late components), as well as the trial-by-trial variability of the unsigned PE regressor from the RL model, while removing the specific trial-to-trial correlations in individual experimental runs. Thus for each resampled iteration and each regressor type, all trials were drawn from the original *y*-value and |PE| distribution, however, the specific values were mixed across trials and runs. In other words, each subject had the same resampled run *y*-values and |PE|'s for a given iteration, though the resulting regressors for each subject were different given that each had a random sequence of regressor amplitude events.

This procedure was repeated 100 times. For each of the 100 resampled iterations, a full 3-level analysis (run, subject and group) was performed. Our design matrix included the same regressors of non-interest used in all our GLM analyses. In turn this allowed us to construct the null hypothesis H0, and establish a joint threshold on cluster size and *Z*-score based on the cluster outputs from the permutated parametric regressors. Specifically, we extracted cluster sizes from all activations exceeding a minimal cluster size (10 voxels) and *Z*-score (2.57 per voxel) for both positive and negative correlations with the permuted parametric regressors. Finally, we examined the distribution of cluster sizes (number of voxels) for the permuted data and found that the largest 5% of cluster sizes exceeded 76 voxels. We therefore used these results to derive a corrected threshold for our statistical maps, which we then applied to the clusters observed in the original data (that is, *Z*=2.57, minimum cluster size of 76 voxels, corrected at *P*=0.05).

### Extracting time-series data

Time-series data from subject-specific clusters of interest were extracted for a PPI (see below). Specifically, we first identified clusters of interest at the group level (that is, in standard space) by applying the cluster correction procedure described above. We subsequently back-projected these clusters from standard space into each individual's EPI (functional) space by applying the inverse transformations as estimated during registration (see fMRI pre-processing section). Each cluster was then checked against the relevant (regressor specific) statistical maps in individual brains (at a slightly more lenient threshold of *P*<0.01 uncorrected, cluster size >10 voxels (90 mm^3^)) to ensure that the inverse-transformation was performed properly. Finally, average regression coefficient or time-series data from all voxels in the back-projected clusters in each subject were computed and normalized for each of the positive and negative regressors.

### PPI analysis

Using the procedure described above, we extracted time-series data from individual clusters in the CM-THAL (bilaterally) of the Early value system, which served as a seed region (that is, physiological regressor—PHY) for a PPI analysis^28^,^69^. This analysis was primarily designed to investigate the potential interaction of the Early and Late systems following negative outcomes. As such, the increase in correlation between the CM-THAL and potential regions of the Late system should be specific for the task in which this coupling is relevant; that is, it should be greater during processing of negative compared with positive outcomes (since the Early system engages only after negative outcomes). Therefore our psychological (PSY) task regressor was constructed such that negative outcomes were weighted +1 and positive outcomes were weighted −1 (using the EEG STV in the Early system to modulate the regressor amplitudes instead yielded identical results, see Supplementary Note 10). The PPI analysis thus included the following regressors during the outcome phase: (1) an unmodulated regressor (all event amplitudes set to 1), (2) the PHY regressor, (3) the PSY regressor and (4) the interaction regressor (PHY × PSY). The rest of the design was identical to GLM 1/2/3. Correction for multiple comparisons was performed on the whole brain using the outcome of the resampling procedure as described earlier. Finally, we note that we used this analysis to also search for increased coupling within the Early system itself following negative outcomes.

### Thalamostriatal connectivity predicting behaviour

To test whether the strength of the connectivity between the CM-THAL and NAcc as identified in our PPI analysis (see PPI analysis section; Fig. 3c) could predict participants' choice behaviour, we performed the following between-subject correlation analyses: we correlated the individual PPI regression coefficients from subject-specific NAcc clusters with (1) the fraction of switch choices away from the symbol that led to a negative outcome (the next time that symbol was offered) and (2) the individual negative learning rates from the RL model (representing individual tendencies to weigh recent negative outcomes more strongly). In addition, to confirm that it was not the activity of the individual regions (CM-THAL and NAcc driving the correlations above), we performed a separate regression analysis. Specifically, in addition to the strength of the thalamostriatal coupling (PPI coefficients), we also included the activity of the CM-THAL and NAcc as separate predictors of switches and negative learning rates, respectively. The results of these analyses are depicted in Fig. 3d in the main text.

## Additional information

**How to cite this article:** Fouragnan, E. *et al.* Two spatiotemporally distinct value systems shape reward-based learning in the human brain. *Nat. Commun.* 6:8107 doi: 10.1038/ncomms9107 (2015).

## Supplementary Material

Supplementary Information Supplementary Figures 1-4, Supplementary Tables 1-5, Supplementary Notes 1-10, and Supplementary Methods 1 and 2
