# Supplementary material for: Two spatiotemporally distinct value systems shape reward-based learning in the human brain
Source: Nat Commun. 2015 Sep 8;6:8107. doi: 10.1038/ncomms9107 (PMC4569710; doi:10.1038/ncomms9107)
Supplement: Supplementary Information — Supplementary Figures 1-4, Supplementary Tables 1-5, Supplementary Notes 1-10, and Supplementary Methods 1 and 2 [file ncomms9107-s1.pdf]

Supplementary Figure 1.

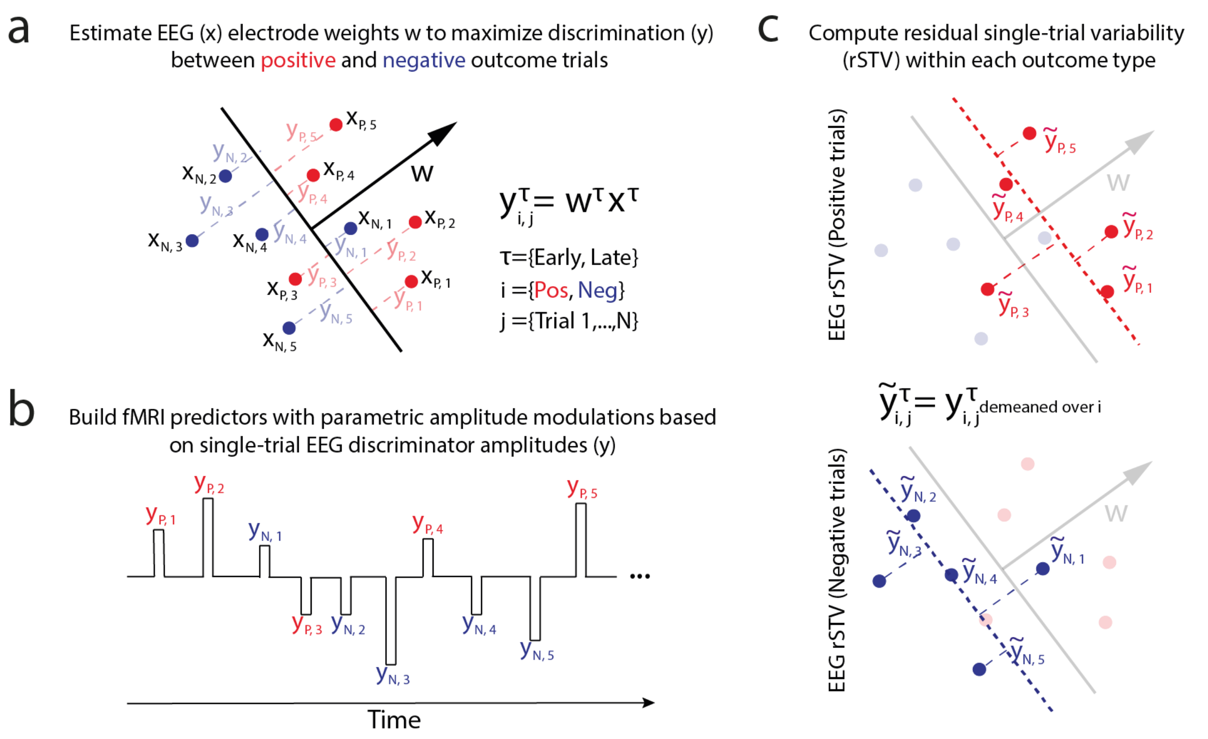

Constructing EEG-informed fMRI regressors. **(a)** For different temporal windows τ (e.g. early and late components) we first estimate $\mathbf{w}$, which is a linear weighting on the EEG sensor data ($\mathbf{X}$) that maximally discriminates between positive (red) and negative (blue) outcome trials. This determines a task-related projection ($\mathbf{y}$) of the data, in which the distance to the decision boundary reflects the decision certainty of the classifier in separating positive and negative outcomes. We treat the single-trial $\mathbf{y}$ amplitudes as an index of how an outcome value is perceived on individual trials. **(b)** Given these $\mathbf{y}$ values and their corresponding outcome-locked onset time points, we build fMRI regressors for the GLM analyses as described in the Methods (GLM2). These regressors are all convolved with the canonical HRF. Details of specific events included in each EEG-informed fMRI regressor can be found in the main text (see fMRI analysis section). **(c)** To create separate EEG-informed regressors for each outcome type, we first estimated the mean discriminator amplitude for each of the positive (left) and negative (right) outcome types (blue and red dotted lines respectively). Then for each outcome type we computed the single-trial residual amplitudes about their corresponding mean response (i.e. the distance of each data point from the outcome-specific mean response). These amplitudes were then used to build separate predictors for positive and negative outcomes for each of the Early and Late components (GLM3).

Supplementary Figure 2.

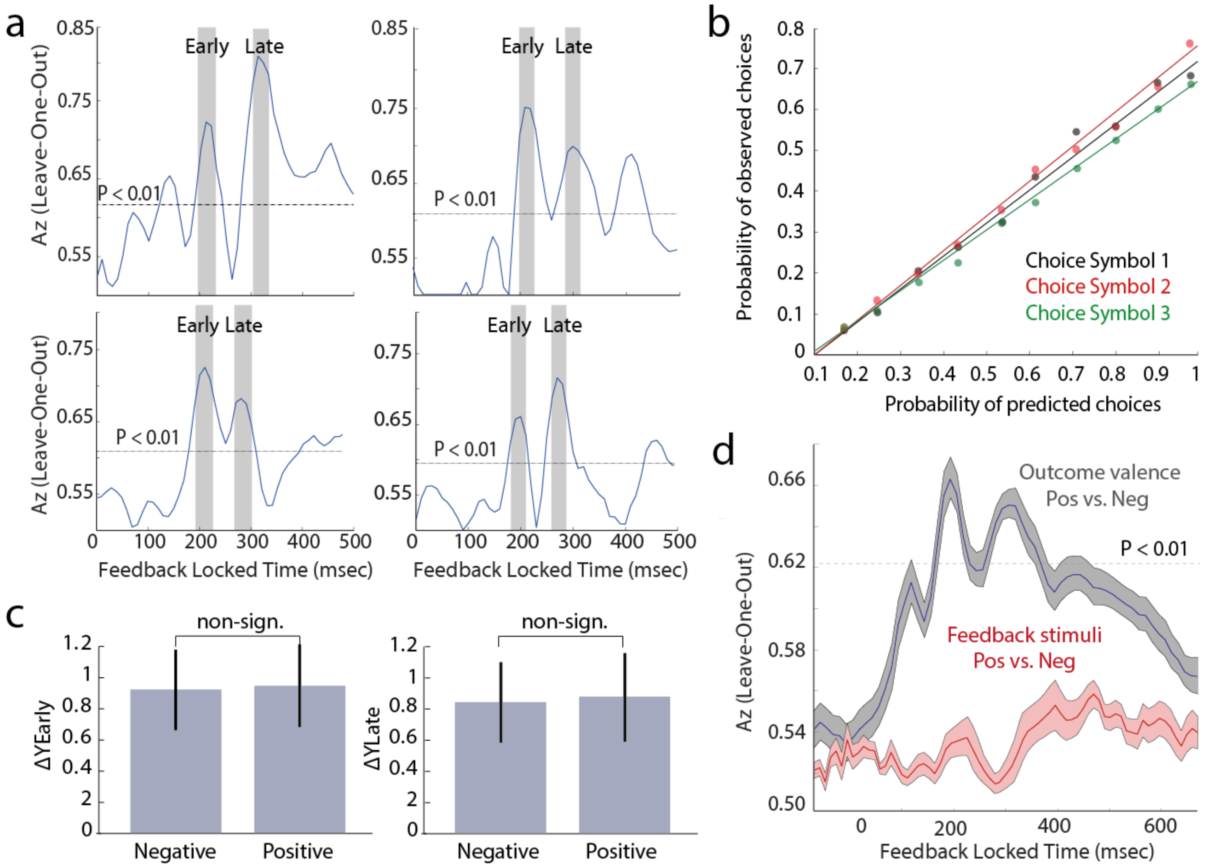

Control analyses. (a) Discriminator performance (Az) during positive-vs-negative outcome discrimination of outcome-locked EEG data, for four representative subjects. The dotted line represents the subject-specific Az value leading to a significance level of $P = 0.01$, estimated using a bootstrap test. The two shaded areas represent the Early and Late windows used for computing the single-trial EEG variability (EEG-STV) in component amplitudes that were used to build EEG-informed BOLD predictors for the fMRI analysis. Individual Early and Late components were present in all participants. These results are consistent with our previous stand-alone EEG findings [16], further confirming our data were of sufficiently high quality after removal of MR-related artifacts. **(b)** Model-predicted choice probabilities (x-axis) derived from a RL algorithm using the softmax procedure (for each symbol separately, binned into 10 bins – bin size of 0.1 - and averaged across all participants) closely matched participant's observed behavioral choices (y-axis), calculated for each bin as the fraction of trials in which participants chose one of the three symbols. **(c)** The relative difference in discriminator output between positive and negative outcomes (for the Early and Late components separately) delivered either in high negative or high positive outcome contexts. No significant differences of context were observed in either component. **(d)** Separate discrimination analyses revealed that the main effects of outcome value during positive-vs-negative outcome discrimination of feedback-locked EEG data (N = 20, in gray) were not driven by the visual properties of the feedback stimuli (N = 7, in red). Discriminator performance (Az-values) of outcome-locked EEG data during passive viewing of the feedback stimuli used in the main task (tick-vs-cross) did not reach significance for any time window. The dotted line represents the group Az-value leading to a significance level of $P < 0.01$ estimated using a bootstrap test.

Supplementary Figure 3.

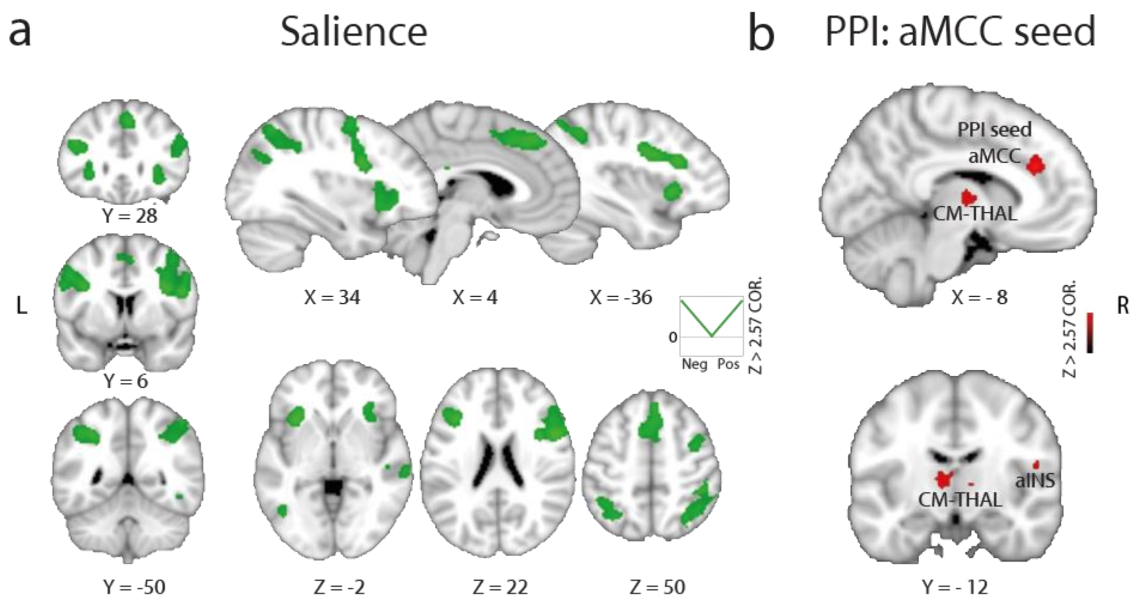

Salience network activations and additional PPI analysis. (a) Activations correlating with a parametric regressor for PE magnitude (i.e. proxy for salience) in a distributed but largely separate network compared to our Early and Late components. Salience signals exhibit a *V*-shape response profile such that activations are stronger for "High negative" and "High positive" PEs. **(b)** The aMCC of the Early system exhibited a positive coupling with other regions of the Early system, namely the CM-THAL and aINS. Using the aINS or THAL as PPI seeds yielded the same findings confirming that these three regions covary together within the Early outcome value system. All activations are rendered on the standard MNI brain at $Z > 2.57$, corrected using a resampling procedure (minimum cluster size = 76 voxels; see Resampling procedure for fMRI thresholding in Methods).

Supplementary Figure 4.

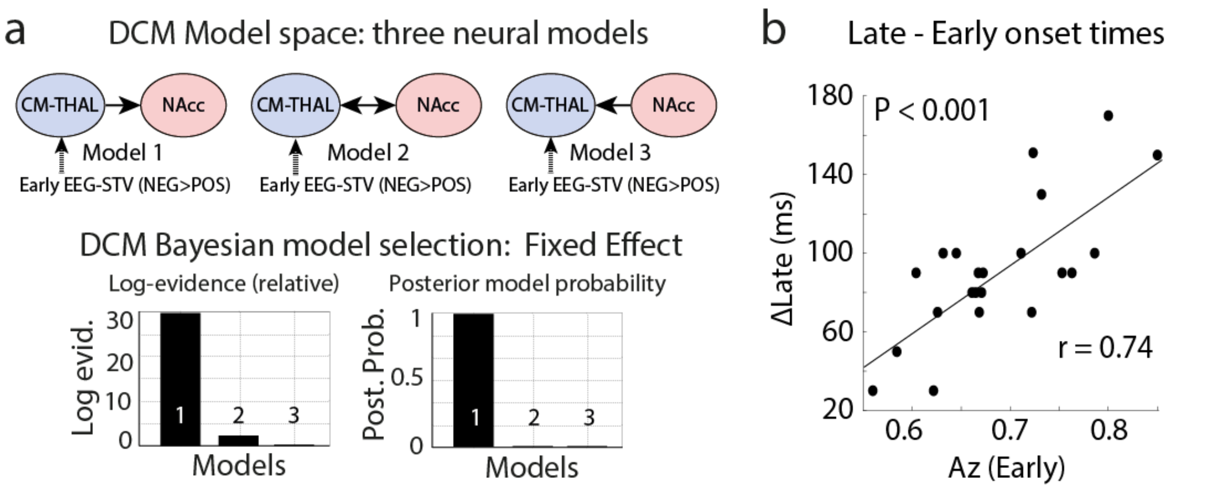

Interaction between Early and Late systems. (a) Top: DCM model space comprised of three different neural models. The driving input represented the main EEG-STV for the Early system as was done for the PPI analysis earlier. Bottom: DCM Bayesian model selection results. Left panel: relative log-evidence for the three models compared using FFX BMS (Fixed-effects Bayesian model selection). Model 1 shows the highest log-evidence relative value (log-evid. = 29). Right panel: model posterior probability exceeds 99% in favor of Model 1. This finding suggests a unilateral connection from CM-THAL to NAcc, consistent with the relative timing of these activations as captured by our EEG-informed fMRI analysis. **(b)** Correlating the strength of the Early EEG component (as quantified by our discriminator's performance – Az value) significantly correlated with the onset times of the Late component (computed relative to the onset of the Early) across participants.

Supplementary Table 1.

Region	Hemisphere	BA	Peak MNI coordinates (mm) *x*	*y*	*z*	Z Value (Peak)
GLM 1						
Conventional Negative > Positive (Z < - 1.67 uncor.)						
Anterior mid-cingulate cortex	R	32	8	22	36	-3.06
	L	32	-6	20	38	-2.36
Supplementary motor area	R	6	14	2	66	-3.73
	L	6	-12	2	66	-3.16
Dorsolateral prefrontal cortex	R	46	38	34	28	-2.1
	L	9/46	-36	38	38	-2.39
Occipital pole	R	18	28	-90	-12	-3.08
	L	18	-20	-96	-2	-4.06
Precuneus	R	7	12	-70	48	-3.08
Conventional Positive > Negative (Z > 2.57 cor.)						
Ventromedial prefrontal cortex	L	10	-4	48	-2	4.32
Striatum (Nucleus Accumbens)	R	-	10	10	8	4.36
	L	-	-8	12	-8	3.56
Amygdala	R	20/34	26	-6	-18	2.72
Dorsal posterior cingulate cortex	R	23	8	-35	28	3.84
Putamen	R	-	27	-8	4	3.81
Posterior insula	L	48	-38	-2	8	4.12
Lateral orbitofrontal cortex	R	47	34	38	-12	3.09
	L	47	-40	38	-12	3.59
Precuneus	R	5	2	-45	56	2.65
Middle temporal gyrus	R	37	48	-60	0	4.42
	L	37	-58	-58	0	3.56
Angular gyrus	R	40	48	-46	54	2.72

Conventional fMRI of outcome value. Complete list of activations showing greater response to Positive > Negative (mixed effects, $Z > 2.57$, corrected) and Negative > Positive (mixed effects, $Z < -1.67$ uncorrected) outcomes, using a categorical outcome valence regressor (GLM 1). MNI, Montreal Neurological Institute; L, left hemisphere; R, right hemisphere, BA, Broadmann Area.

Supplementary Table 2.

Region	Hemisphere	BA	Peak MNI coordinates (mm)			Z Value (Peak)
			x	*y*	*z*	
GLM 2						
Positive correlation with early STV-EEG						
Non-significant						
Negative correlation with early STV-EEG (Z < - 2.57 cor.)						
Anterior mid-cingulate cortex	R	24	4	16	34	-3.78
	L	24	-8	20	30	-3.71
Supplementary motor area	R	6	8	0	68	-3.91
	L	6	-6	8	48	-3.42
Dorsolateral prefrontal cortex	R	9/46	32	36	36	-3.52
	L	46	-38	26	38	-3.97
Centromedial thalamus	R	-	10	-12	8	-3.13
	L	-	-10	-16	8	-3.01
Anterior insula	R	48	38	12	2	-2.98
	L	48	-40	14	-4	-2.76
Posterior mid-cingulate / Dorsal posterior cingulate cortex	R	23	4	-24	42	-3.13
Inferior lateral orbitofrontal cortex	R	38/47	52	24	-2	-3.08
	L	47	-36	30	-2	-2.99
Dorsomedial prefrontal cortex	L	9/10	-8	58	32	-3.46
Occipital pole	R	18	24	-94	-14	-3.43
	L	17	-4	-98	-2	-3.01
Precuneus	L	7	-2	-74	48	-3.06
	L	7	-4	-54	50	-3.29
Angular gyrus	R	21/22	48	-50	20	-3.09
	L	21	-58	-56	20	-2.76
Lingual gyrus	L	17	-6	-76	4	-2.67
Positive correlation with late STV-EEG (Z > 2.57 cor.)						
Ventromedial prefrontal cortex	L	10	-6	52	6	3.84
Striatum (Nucleus Accumbens)	R	-	8	10	-6	3.55
	L	-	-8	10	-8	3.58
Amygdala	R	28/36	26	-2	-22	2.71
	L	28/34	-20	-4	-18	3.63
Dorsal posterior cingulate cortex	L	23	-8	-38	32	3.54
Ventral posterior cingulate cortex	L	29	-2	-50	20	3.27
Anteromedial prefrontal cortex	L	32	-8	50	30	3.51
Superior medial prefrontal cortex	L	8	-12	28	60	3.48
Postcentral gyrus	R	43	62	-12	28	3.32
	L	43	-56	-12	28	3.46
Putamen	R	-	30	-6	2	3.66
	L	-	-28	-6	2	3.52
Posterior insula	L	48	-40	-2	8	2.98
Lateral orbitofrontal cortex	R	38	34	18	-18	3.05
	L	38/48	-24	12	-20	2.81
Anterior cingulate cortex	R	24	4	34	16	3.2
Precuneus	L	-	-10	-56	30	3.56
	L	30	-6	-56	14	3.13
Middle temporal gyrus	R	20/37	56	-46	-8	2.85
	L	37	-64	-48	-6	2.78
Angular gyrus	R	39	44	-66	34	4.07
	L	39	-50	-64	40	2.93
Negative correlation with late STV-EEG						
Non significant						

EEG-informed fMRI reveals Early and Late value systems. Complete list of activations correlating either positively or negatively with the single-trial variability in the Early and Late EEG components (GLM 2; mixed effects, |*Z*| > 2.57, corrected). MNI, Montreal Neurological Institute; L, left hemisphere; R, right hemisphere, BA, Broadmann Area.

Supplementary Table 3.

Region	Hemisphere	BA	Peak MNI coordinates (mm) *x*	*y*	*z*	Z Value (Peak)
GLM 3						
Early-negative outcome STV-EEG (Z < - 2.57 cor.)						
Anterior mid-cingulate cortex	R	32	8	22	34	-4.14
	L	24	-2	26	28	-3.29
Supplementary motor area	R	6	10	0	66	-3.68
	L	6	-6	2	48	-2.82
Dorsolateral prefrontal cortex	R	46	32	34	32	-3.17
	L	9/46	-26	36	32	-3.11
Centromedial thalamus	R	-	10	-10	12	-3.32
Anterior insula	R	48	36	14	4	-2.79
	L	48	-40	14	-4	-2.78
Posterior mid-cingulate / Dorsal posterior cingulate cortex	R	23	2	-26	42	-2.99
Inferior lateral orbitofrontal cortex	R	38/47	48	22	-6	-2.69
	L	38	-54	14	-6	-3.4
Dorsomedial prefrontal cortex	L	10	-10	56	28	-2.62
Occipital pole	R	18	12	-92	-8	-2.63
Precuneus	L	-	-10	-50	46	-2.57
Angular gyrus	R	22/42	56	-46	20	-3.37
	L	22/39	-58	-54	32	-2.69
Early-positive outcome STV-EEG (Z < - 2.57 cor.)						
Dorsolateral prefrontal cortex	L	45/46	-42	34	32	-3.51
Late-negative outcome STV-EEG (Z > 2.57 cor.)						
Ventromedial prefrontal cortex	L	10/11	-2	42	4	2.69
Striatum (Nucleus accumbens)	R	-	6	10	-6	3.42
	L	-	-6	8	-6	2.97
Amygdala	L	28	-24	-8	-18	3.01
Dorsal posterior cingulate cortex	-	23	0	-28	40	3.08
Anteromedial prefrontal cortex	L	10	-6	62	8	3.2
Superior medial prefrontal cortex	L	9	-6	46	32	3.64
Putamen	R	-	30	-6	4	3.43
	L	-	-26	0	4	2.92
Lateral orbitofrontal cortex	R	11	26	34	-12	2.78
	L	47	-30	24	-12	2.78
Anterior cingulate cortex	R	32	10	42	14	3.36
Middle temporal gyrus	R	37	56	-50	-8	2.8
	L	37	-60	-54	-4	3.09
Angular gyrus	L	39	-46	-56	38	2.98
Late-positive outcome STV-EEG (Z > 2.57 cor.)						
Ventromedial prefrontal cortex	-	10	0	52	-2	2.85
Striatum (Nucleus accumbens)	R	-	6	14	-4	2.96
Amygdala	L	34	-18	-2	-16	2.71
Dorsal posterior cingulate cortex	L	23	-4	-18	38	3.21
Ventral posterior cingulate cortex	L	29	-3	-50	18	3.45
Anteromedial prefrontal cortex	L	10	-4	60	14	3.14
Superior medial prefrontal cortex	-	9	0	52	32	3.01
Postcentral gyrus	R	43	60	-12	30	2.61
	L	43	-60	-10	30	3.03
Putamen	R	-	30	-4	-4	2.99
	L	-	-18	6	-8	3.03
Posterior insula	L	48	-42	-8	6	2.59
Precuneus	L	17	-4	-58	12	3.37
Angular gyrus	L	37/39	-42	-60	16	2.76

Early and Late responses to positive and negative outcomes. Complete list of activations correlating with the trial-to-trial *residual* fluctuations in the Early and Late EEG components computed separately for positive and negative outcomes (GLM 3; mixed effects, $|Z| > 2.57$, corrected); MNI, Montreal Neurological Institute; L, left hemisphere; R, right hemisphere, BA, Broadmann.

Supplementary Table 4.

	Early		Late	
	Positive	Negative	Positive	Negative
Regions of the Early system				
Anterior mid-cingulate cortex		✓		
Supplementary motor area		✓		
Dorsolateral prefrontal cortex	✓	✓		
Centromedial thalamus		✓		
Anterior insula		✓		
Posterior mid-cingulate / Dorsal posterior cingulate		✓		
Inferior lateral orbitofrontal cortex		✓		
Dorsomedial prefrontal cortex		✓		
Occipital pole		✓		
Precuneus		✓		
Angular gyrus		✓		
Lingual gyrus				
Regions of the Late system				
Ventromedial prefrontal cortex			✓	✓
Striatum (Nucleus Accumbens)			✓	✓
Amygdala			✓	✓
Dorsal posterior cingulate cortex			✓	✓
Ventral posterior cingulate cortex			✓	
Anteromedial prefrontal cortex			✓	✓
Superior medial prefrontal cortex			✓	✓
Postcentral gyrus			✓	
Putamen			✓	✓
Posterior insula			✓	
Lateral orbitofrontal cortex				✓
Anterior cingulate cortex				✓
Precuneus			✓	
Middle temporal gyrus				✓
Angular gyrus			✓	✓

Distribution of regions in responses to positive and negative outcomes. Distribution of regions of the Early and Late systems showing the extent to which positive and negative outcomes could separately explain the BOLD responses associated with each of the two systems.

Supplementary Table 5.

Region	Hemisphere	BA	Peak MNI coordinates (mm)			Z Value Peak)
			x	*y*	*z*	
Salience |PE| (Z > 2.57 cor.)						
Middle frontal gyrus	R	44	48	12	34	3.25
	L	44	-50	8	38	3.49
Anterior insula	R	48	34	18	0	3.13
	L	48	-36	20	0	4.02
Supplementary motor area	R	8	2	20	52	3.76
Middle temporal gyrus	R	21	62	-26	-6	3.28
Inferior frontal gyrus	R	44	52	10	18	3.69
Inferior temporal gyrus	L	37	-40	-60	-10	3.73
Supramarginal gyrus	R	40	40	-38	38	3.41
	L	40	-40	-48	42	3.46
Precentral gyrus	R	6/44	38	4	34	3.41
	L	6	-52	0	34	3.61
Angular gyrus	R	40	40	-48	40	3.48
	L	40	-46	-54	42	3.41

FMRI responses to outcome salience. Complete list of activations correlating positively with the outcome salience (unsigned PE signal), estimated with a classical RL model (mixed effects, Z > 2.57, corrected); MNI, Montreal Neurological Institute; L, left hemisphere; R, right hemisphere, BA, Broadmann.

Supplementary Note 1. Behavioral performance. Participants performed 340 trials during the course of the experiment separated in 2 blocks and experienced on average 176.75 (±7.66) negative and 149.85 (±7.25) positive outcomes. Overall they achieved 20.4 reversals (± 2.1) with an average of 12.7 trials (± 2.2) before reaching the learning criterion (16.4 ± 2.2 including the buffer trials) suggesting a high level of engagement and accurate representation of the task.

Supplementary Note 2. Outcome value in individual EEG data. Using single-trial multivariate discriminant analysis on outcome-locked EEG responses, we identified two temporally distinct EEG components discriminating between positive and negative outcomes; on average, at 219ms (± 26.5) (Early component) and 308ms (± 37.7) (Late component) following the outcome. We found this pattern of discriminator performance in all subjects (Supplementary Fig. 2a for individual subject data), highlighting the robustness of these effects across individual participants.

Supplementary Note 3. Controlling for salience. To further establish that our Early and Late EEG-derived components encode the value of the outcome rather than salience or uncertainty effects, which we quantified as the amount to which outcomes deviated from expectations (i.e. unsigned PE in the RL model), we performed an additional control analysis. Specifically, we correlated the single-trial EEG component amplitudes with the unsigned PE estimates resulting from (Supplementary Equation (1)). Specifically, we performed separate regression analyses using the single-trial discriminator amplitude values derived for the Early and Late components, to predict the single-trial unsigned PE estimates resulting from the RL model. We repeated this analysis separately for positive and negative outcomes and tested whether the resulting regression coefficients came from a distribution with mean different than zero (using a two-tailed t-test). Crucially*,* we found that neither the Early nor the Late outcome value components were correlated with the RL model's unsigned PE estimates (Early, negative outcomes: t_{19} = 0.41, P = 0.68; Late, negative outcomes: t_{19} = 1.54, P = 0.2; Early, positive outcomes: t_{19} = 0.9, P = 0.37; Late, positive outcomes: t_{19} = 1.09, P = 0.29), confirming that our EEG component amplitudes do not reflect unsigned PE's but rather encode outcome valence alone.

Note that, we also included the single-trial PE magnitude estimates from the RL model as a parametric regressor in all fMRI analyses to similarly control for salience effects in the functional data. The observed activations (i.e. human salience network - see 'Salience fMRI

results' section below) further confirmed that our unsigned PE estimates properly controlled for salience.

Supplementary Note 4. Controlling for outcome context. We also ran another control analysis to ensure that the Early and Late EEG-derived outcome components were not merely driven by the context in which the outcome was delivered. For example, after experiencing many negative outcomes, a positive feedback could draw more attentional resources and result in enhanced perceptual and/or cognitive processing of that outcome as compare to a negative one (and vice versa for negative outcomes within a positive context) [7,8]. In turn this could result in differences in activity between positive and negative outcomes, which could explain our discriminator's performance at either of the Early and Late time windows.

To mitigate this concern we performed an additional analysis in which we first determined the context in which each trial was delivered by considering the outcome on the 20 trials preceding the one under consideration. Specifically, each trial was categorized as "-1" if there were more negative than positive outcomes during the previous 20 trials and "+1" otherwise. Having divided the trials up into the two contexts, we subsequently computed the difference in discriminator amplitudes between positive and negative outcome trials ($y_{diff}(\tau)$ = $y_{pos}(\tau) - y_{neg}(\tau)$) for each context type. We expected to find differences in $y_{diff}(\tau)$ between the two context types if the observed discriminator differences where driven by one of the context types. We formally tested for this using a paired t-test on the estimated $y_{diff}(\tau)$ across the two context types and separately for each discriminator component (Early and Late). Crucially, we found that neither the Early nor the Late outcome value components were driven by the context in which the outcome was delivered (Early: t_{19} = 0.33, P = 0.743 for the Early. Late: t_{19} = 0.43, P = 0.667; Supplementary Fig. 2c). We repeated this analysis using different trial bin sizes (5-30 in increments of 5 trials) and found no significant effects of outcome context (all P > 0.34).

Supplementary Note 5. Controlling for visual properties of the stimuli. To ensure that low-level visual features associated with our feedback stimuli (tick vs cross) could not account for the Early and Late EEG components we ran a separate experiment. Specifically, we performed a stand-alone EEG experiment in which seven participants (mean age = 28, ± 3.46) were instructed to passively view each of the two feedback stimuli used in the main task presented in random order. We recorded EEG data from the same MR-compatible caps used for the main experiment inside the scanner. The stimuli were presented for 650 ms (to match the stimulus duration used in the main experiment) and the inter-stimulus interval

varied between 2 and 4 seconds. Pre-preprocessing of the EEG was identical to that used for the main experiment. Similarly, we ran a single-trial multivariate discriminant analysis [5,6] to estimate linear spatial weightings of the EEG sensors that discriminated between the visual stimuli representing positive and negative feedback. The discrimination results clearly demonstrate that the effects we observed in the main task could not be purely explained by the visual properties of the stimuli used to deliver feedback to participants (Supplementary Fig. 2d).

Supplementary Note 6. Salience fMRI responses. Activity in several areas of the human salience network [9,10], including the dorsolateral prefrontal cortex, dorsal mid-cingulate cortex, precentral gyrus, anterior insula, and middle temporal gyrus (Supplementary Fig. 3, Supplementary Table 5) correlated positively with a salience measure derived from the magnitude of the PE signal ($Z > 2.57$, $P < 0.05$ corrected, minimum voxel cluster = 76). These findings further confirmed that our unsigned PE estimates from a simple RL model properly accounted for salience effects. No areas exhibited negative correlation with the modulus of the PE signal.

Supplementary Note 7. EEG components predict behavioral responses. To establish a more concrete link between our Early and Late EEG components and behavior, we first performed a logistic regression analysis to test the extent to which the single-trial amplitudes of the two components on the current trial were predictive of behavioral switching on the next trial. We found that both components were predictive of participants' switch patterns (Early: $t_{19} = -2.75$, $P = 0.012$; Late: $t_{19} = -3.17$, $P = 0.005$) consistent with previous reports. More specifically, we found that the more negatively an outcome was encoded (more negative discriminator amplitudes) the higher the likelihood of a switch (or conversely, the more positively an outcome was encoded as indexed by more positive discriminator amplitudes the likelihood of choosing the same symbol again in the next increased)

In addition we performed two separate analyses to associate each of the Early and Late components specifically with their hypothesized roles in early automatic response and reward/value processing respectively. We tested whether the discriminator amplitudes associated with our Early and Late EEG components following negative outcome trials could be used to predict response slowing in the subsequent decision (i.e. as indexed by difference in RTs between the current and next trial). Here we used a linear regression analysis and found that the Early ($t_{19} = -2.39$, $P = 0.027$), but not the Late ($t_{19} = 1.44$, $P = 0.17$), component was predictive of response caution. Specifically, we found that the more negatively an outcome was encoded (more negative discriminator amplitudes) the longer it took for

participants to respond on the next trial. This finding is consistent with the hypothesized role of the early system in alertness and avoidance behavior.

Finally, we tested whether the discriminator amplitudes associated with our Early and Late EEG components were predictive of the value updating on the chosen symbol. Once again, we used a linear regression analysis and found that the Late (t_{19} = 2.13, P = 0.04), but not the Early (t_{19} = 1.12, P = 0.27), component was predictive of the value update on the chosen option (as estimated with our RL model). Specifically, we found a positive association between the degree of up- and down-regulation of expected value and the amplitude of the late EEG component – the more positive the late discriminator amplitudes, the higher the likelihood that the chosen value would increase relative to its previous estimate. Correspondingly the more negative the late discriminator amplitudes, the higher the likelihood that the chosen value would decrease. This finding is consistent with the hypothesized role of the Late system in reward processing and the up/down regulation of value information. Please note that in all regressions we used a separate predictor for the unsigned PE (e.g. as estimated by our RL model) to be consistent with our fMRI GLMs and to account for potential effects of outcome salience.

Supplementary Note 8. Early system predicting onset time of the Late system in the EEG. Our connectivity analyses provided evidence that the Early value system serves a dual role: 1) mediating an initial alertness response and 2) down-regulating the response profile of the Late system following negative outcomes to promote avoidance learning. It stands to reason that the strength of the initial alertness response might have consequences on the timing of the interaction with the Late system. In other words, if the initial response to the negative outcomes is stronger (i.e. to act to avoid a thread) the interaction with the Late system to update future expectations (i.e. apply a learning rule) should be delayed accordingly.

Correspondingly, in the EEG we observed significant variability in the onset time of the Late component. We therefore performed a between subject correlation comparing the strength of the Early component (individual Early component Az values) with the onset time of the Late component (relative to the Early). We found a highly significant correlation between the two measures (r = 0.74, P < 0.0001; Supplementary Fig. 4b) indicating that those participants exhibiting strong activations in the Early component postponed reward-related learning processing as seen in the Late system by a corresponding amount. This finding also highlights the interplay between the two outcome value systems that we have already established with our connectivity analysis.

Supplementary Note 9. Control for shared variance in GLM2. To account for the shared variance between our EEG-informed regressors in GLM2, we also performed two additional analyses. Specifically, we repeated GLM2 while orthogonalizing the regressor for the Early EEG component with respect to the one for the Late EEG component and vice versa. We found that in both designs the activations correlating with the Early and Late components remained identical to those in the original GLM2. We view this as additional evidence that it is the trial-by-trial variability in these component amplitudes that helps tease these activations apart rather than the categorical difference between positive and negative outcomes as such.

Supplementary Note 10. Additional PPI analyses. We employed an identical PPI procedure as the one presented in our main text (see Methods PPI analysis section) using either the aMCC or aINS (as identified in GLM 2 within the Early system) as seed regions (i.e. PHY regressor). These analyses were designed: 1) to investigate whether there are functional connections *within* the Early system itself and 2) to test whether in addition to the CM-THAL there are other regions which interact with the Late system following negative outcomes. Using either the aMCC or aINS as seed confirmed the original findings of positive coupling of the CM-THAL, aMCC and aINS within the Early system. However, neither the aMCC nor the aINS showed a significant inverse coupling with the Late system as was found for the CM-THAL in the main text.

SUPPLEMENTARY METHODS

Reinforcement learning algorithms. We used a reinforcement-learning (RL) algorithm to estimate trial-by-trial prediction errors (PEs) using each subject's behavioral responses [1]. Because we were interested in potential differences between positive and negative outcomes, we fit behavioral data using separate learning rate parameters for each outcome (α_{pos} and α_{neg}) as was done in [2,3]. Specifically, if stimulus *A* was selected on trial *i*, its value was updated via a PE, δ, as follows:

$$v_A(i+1) = v_A(i) + \alpha_{pos}.\theta.\delta(i) + \alpha_{neg}.(1-\theta).\delta(i) \quad (1)$$

where θ is set to 1 for positive outcomes and to 0 for negative outcomes such that the learning rates α_{pos} and α_{neg} depend on whether the outcome is better or worse than expected. The PE was given by $\delta(i) = r(i) - v_A(i)$. In contrast, the values of the unselected stimulus (e.g. *B*) and the stimulus that was not shown on that trial (e.g. *C*) were not updated. To generate choices, we first used a softmax procedure in which, on every trial, the probability of choosing stimulus *A* was given by:

$$P_A(i) = \sigma(\beta(v_A(i) - v_B(i)) - \phi) \quad (2)$$

where $\sigma(z) = 1/(1+e^{-z})$ is the logistic function, ϕ denotes the indecision point (at which both stimuli were chosen with equal probability) and β the degree of stochasticity in making the decision. The model choice probabilities were then fitted against the discrete behavioral choices to estimate the free parameters (α_{pos}, α_{neg}, β, ϕ). This was done using maximum likelihood estimation and a constrained non-linear optimization procedure (as implemented in *fmincon* in MATLAB) separately for each subject. The associated likelihood function was given by:

$$logL = \frac{\sum B_A logP_A + \sum B_B logP_B}{N_A + N_B} \quad (3)$$

where N_A and N_B denote the number of trials in which stimulus A and B were chosen, and B_A (B_B) equals 1 if A (*B*) was chosen on that trial, and 0 otherwise. We fitted this function similarly for the other two stimulus combinations (*AC* and *BC*) and found the optimal parameters by minimizing the sum of the three negative log-likelihoods.

To visualize the goodness of fit we compared the choice probabilities predicted by the RL model using the softmax procedure to subjects' behavioral choices by binning P (Supplementary Equation (2)) into 10 bins (bin size of 0.1) and calculating for each bin the fraction of trials in which subjects chose one stimulus (Supplementary Fig. 2b). Interestingly, this result also indicates that when participants were selecting between the two less rewarding symbols, they chose the one that carried the highest expected value based on previous history.

Finally, we also fit our data with a version of the RL model in which the update of unchosen item values was anti-correlated with that of the chosen option as in Gläscher et al. [4]. The resulting learning rates and PEs were highly correlated with those obtained with our original model ($r = 0.98$ for the PEs and $r = 0.76$ for the learning rates). Repeating the correlations of the strength of the thalamostriatal coupling with the new learning rates (as in Fig. 3d) yielded identical results ($r = -0.74$; $P < 0.001$). Overall, direct model comparison using a Bayesian Information Criterion revealed that our original model was a better predictor of behavior.

Dynamic Causal Modeling. To provide additional evidence for the causal relationship between the two regions identified in our main PPI analysis, namely the CM-THAL and the NAcc, we applied dynamic causal modelling (DCM) on our data using the DCM10 toolbox for SPM12 [11,12]. Given the correlation between the CM-THAL activity and the Early STV and the principles governing DCM [13], the DCM analysis was carried out in several steps. We first entered the full GLM2 into SPM12 to generate a design matrix and extracted activation time courses for the two regions of interest. Then we constructed a model space of three neural models that could capture the relationship between the two areas: 1: CM-THAL → NAcc, 2: NAcc → CM-THAL and 3: CM-THAL ↔ NAcc. We used the STV of the Early component as the driving input onto the CM-THAL in all three neural models (see Supplementary Fig. 4a). Finally, we identified the model showing the most likely configuration of connections based on the data, assuming an optimal model structure to be identical across subjects. We thus used fixed-effect (FFX) Bayesian model selection (BMS) that estimates the probability of the data given a particular neural model (e.g. an approximation to the model evidence).

Under the FFX assumption, we computed group log-evidence for each model which corresponds to the sum of log-evidences across subjects [12,14] and found that Model 1 (CM-THAL → NAcc) exceeded the two other models by a difference of 29. Note that, a difference in log-evidence above 3 is taken as strong evidence [15] because the corresponding Bayes factor of exp(3) is about twenty (c.f. the $P < 0.05$ criterion often employed in classical inference). In addition, we showed that the model posterior probability exceeds 99% in favor

of Model 1 (Supplementary Fig. 4a) and suggests a unilateral connection from CM-THAL to NAcc with a driving input onto the CM-THAL. This result is consistent with the relative timing of these activations revealed by our EEG-informed fMRI analysis and supports the thalamostriatal projection found in our PPI analyses.

SUPPLEMENTARY REFERENCES

1. Sutton, R. *Reinforcement Learning: An Introduction*. (MIT Press, 1998).
2. Frank, M. J. Dynamic dopamine modulation in the basal ganglia: a neurocomputational account of cognitive deficits in medicated and nonmedicated Parkinsonism. *J. Cogn. Neurosci.* **17,** 51–72 (2005).
3. Frank, M. J., Moustafa, A. A., Haughey, H. M., Curran, T. & Hutchison, K. E. Genetic triple dissociation reveals multiple roles for dopamine in reinforcement learning. *Proc. Natl. Acad. Sci.* **104,** 16311–16316 (2007).
4. Gläscher, J., Daw, N., Dayan, P. & O'Doherty, J. P. States versus rewards: dissociable neural prediction error signals underlying model-based and model-free reinforcement learning. *Neuron* **66,** 585–595 (2010).
5. Parra, L. *et al.* Linear spatial integration for single-trial detection in encephalography. *NeuroImage* **17,** 223–230 (2002).
6. Parra, L. C., Spence, C. D., Gerson, A. D. & Sajda, P. Recipes for the linear analysis of EEG. *NeuroImage* **28,** 326–341 (2005).
7. Tobler, P. N., Fiorillo, C. D. & Schultz, W. Adaptive Coding of Reward Value by Dopamine Neurons. *Science* **307,** 1642–1645 (2005).
8. Kobayashi, S. & Schultz, W. Reward contexts extend dopamine signals to unrewarded stimuli. *Curr. Biol. CB* **24,** 56–62 (2014).
9. Corbetta, M. & Shulman, G. L. Control of goal-directed and stimulus-driven attention in the brain. *Nat. Rev. Neurosci.* **3,** 201–215 (2002).
10. Pessoa, L., Kastner, S. & Ungerleider, L. G. Neuroimaging Studies of Attention: From Modulation of Sensory Processing to Top-Down Control. *J. Neurosci.* **23,** 3990–3998 (2003).
11. Friston, K. J., Harrison, L. & Penny, W. Dynamic causal modelling. *NeuroImage* **19,** 1273–1302 (2003).
12. Stephan, K. E. *et al.* Dynamic causal models of neural system dynamics:current state and future extensions. *J. Biosci.* **32,** 129–144 (2007).
13. Stephan, K. E. *et al.* Ten simple rules for dynamic causal modeling. *NeuroImage* **49,** 3099–3109 (2010).
14. Garrido, M. I. *et al.* Repetition suppression and plasticity in the human brain. *NeuroImage* **48,** 269–279 (2009).
15. Raftery, A. E. Bayes Factors and BIC Comment on 'A Critique of the Bayesian Information Criterion for Model Selection'. *Sociol. Methods Res.* **27,** 411–427 (1999).

16. Philiastides, M. G., Biele, G., Vavatzanidis, N., Kazzer, P. & Heekeren, H. R. Temporal dynamics of prediction error processing during reward-based decision making. *NeuroImage* **53,** 221–232 (2010).
